# Analyzing the genomic and transcriptomic architecture of milk traits in Murciano-Granadina goats

**DOI:** 10.1186/s40104-020-00435-4

**Published:** 2020-03-11

**Authors:** Dailu Guan, Vincenzo Landi, María Gracia Luigi-Sierra, Juan Vicente Delgado, Xavier Such, Anna Castelló, Betlem Cabrera, Emilio Mármol-Sánchez, Javier Fernández-Alvarez, José Luis Ruiz de la Torre Casañas, Amparo Martínez, Jordi Jordana, Marcel Amills

**Affiliations:** 1 Centre for Research in Agricultural Genomics (CRAG), CSIC-IRTA-UAB-UB, Universitat Autònoma de Barcelona, 08193 Bellaterra, Spain; 2 Departamento de Genética, Universidad de Córdoba, 14071 Córdoba, Spain; 3 Departament de Ciència Animal i dels Aliments, Facultat de Veterinària, Universitat Autònoma de Barcelona, 08193 Bellaterra, Spain; 4 Asociación Nacional de Criadores de Caprino de Raza Murciano-Granadina (CAPRIGRAN), 18340 Granada, Spain; 5 Servei de Granges i Camps Experimentals, Universitat Autònoma de Barcelona, 08193 Bellaterra, Spain

**Keywords:** Casein genes, Dairy traits, GWAS, Lactation, QTLs, RNA-Seq

## Abstract

**Background:**

In this study, we aimed to investigate the molecular basis of lactation as well as to identify the genetic factors that influence milk yield and composition in goats. To achieve these two goals, we have analyzed how the mRNA profile of the mammary gland changes in seven Murciano-Granadina goats at each of three different time points, i.e. 78 d (T1, early lactation), 216 d (T2, late lactation) and 285 d (T3, dry period) after parturition. Moreover, we have performed a genome-wide association study (GWAS) for seven dairy traits recorded in the 1st lactation of 822 Murciano-Granadina goats.

**Results:**

The expression profiles of the mammary gland in the early (T1) and late (T2) lactation were quite similar (42 differentially expressed genes), while strong transcriptomic differences (more than one thousand differentially expressed genes) were observed between the lactating (T1/T2) and non-lactating (T3) mammary glands. A large number of differentially expressed genes were involved in pathways related with the biosynthesis of amino acids, cholesterol, triglycerides and steroids as well as with glycerophospholipid metabolism, adipocytokine signaling, lipid binding, regulation of ion transmembrane transport, calcium ion binding, metalloendopeptidase activity and complement and coagulation cascades. With regard to the second goal of the study, the performance of the GWAS allowed us to detect 24 quantitative trait loci (QTLs), including three genome-wide significant associations: QTL1 (chromosome 2, 130.72-131.01 Mb) for lactose percentage, QTL6 (chromosome 6, 78.90-93.48 Mb) for protein percentage and QTL17 (chromosome 17, 11.20 Mb) for both protein and dry matter percentages. Interestingly, QTL6 shows positional coincidence with the casein genes, which encode 80% of milk proteins.

**Conclusions:**

The abrogation of lactation involves dramatic changes in the expression of genes participating in a broad array of physiological processes such as protein, lipid and carbohydrate metabolism, calcium homeostasis, cell death and tissue remodeling, as well as immunity. We also conclude that genetic variation at the casein genes has a major impact on the milk protein content of Murciano-Granadina goats.

## Background

Understanding how genetic variation shapes the phenotypic diversity of milk traits not only implies the identification of such genetic determinants through genome-wide association studies (GWAS), but also a detailed knowledge about the genes playing a fundamental role in the progression of lactation. So far, very few GWAS have uncovered the genomic location and distribution of polymorphisms affecting milk yield and composition in goats. Martin et al. [1] genotyped, with the Goat SNP50 BeadChip, 2,209 Alpine and Saanen goats and performed association analyses with five dairy traits. Such work enabled the identification of 109 significant associations and further uncovered two polymorphisms in the *DGAT1* gene that have major effects on fat content by modifying the activity of this enzyme [1]. In another recent study, Mucha et al. [2] detected a single nucleotide polymorphism (SNP) on goat chromosome 19 displaying a genome-wide significant association with milk yield as well as a number of chromosome-wide significant associations with dairy traits on chromosomes 4, 8, 14, and 29. Although these two studies represent a valuable step towards elucidating the genomic architecture of milk yield and composition traits in goats, analyzing a broader array of goat populations, as has been done in cattle [3], would provide a more comprehensive view of the genetic determinism of caprine dairy phenotypes.

The events promoting the initiation, maintenance and abrogation of lactation have been barely analyzed from a transcriptomic perspective in goats. Only one RNA-Seq study has investigated the changes experienced by the caprine mammary gland transcriptome across the production cycle (lactation vs. dry period) [4], while another one has compared the gene expression profile of goat milk somatic cells in colostrum and mature milk [5]. A third study investigated the transcriptomes of goat somatic cells, milk fat globules and blood cells via using microarrays [6]. This situation contrasts strongly with that of cattle, in which several studies outlining how the gene expression profile of the mammary gland changes in response to different experimental conditions have been published so far [7–10]. Indeed, RNA-Seq studies performed in dairy cattle [11] and also in sheep [12] have revealed that hundreds of genes are differentially expressed (DE) in the mammary gland when lactating vs. non-lactating individuals are compared. Multiple lines of evidence indicate that many of these genes are related to mammary gland development, protein and lipid metabolism processes, signal transduction, differentiation and immune function, being very significant the downregulation of the protein and lipid biosynthetic machinery [11, 12].

The work presented here had two main objectives: 1) Elucidating the changes in the mammary transcriptome associated with the lactation stage by sequencing total RNA from mammary gland biopsies retrieved from seven Murciano-Granadina goats sampled at 78 d (early lactation), 216 d (late lactation) and 285 d (dry period) post-partum, and 2) Identifying the genetic determinants of milk yield and composition traits in Murciano-Granadina goats through a GWAS approach comprising 822 individuals with records for 7 dairy traits registered during their 1^st^ lactation.

## Methods

### Sequencing the mammary gland transcriptome along the lactation stage

#### Transcriptome sequencing

Mammary biopsies were retrieved from 7 Murciano-Granadina goats at each of the three time points, i.e. 78.25 ± 9.29 d (T1, early lactation), 216.25 ± 9.29 d (T2, late lactation) and 285.25 ± 9.29 d (T3, dry period) after parturition (Additional file 1: Table S1). The average age of the sampled goats was 5.88 ± 1.89 years and none of them was pregnant at T1, T2 or T3 (Additional file 1: Table S1). Mammary tissue was extracted with SPEEDYBELL 14G 150 mm semi-automatic biopsy needles (EVEREST Veterinary Technology, Barcelona, Spain) after applying local anesthesia to the region to be punctured. Samples were immediately submerged in RNAlater stabilization solution (Thermo Fisher Scientific, Barcelona, Spain) and shipped back to the laboratory for storage at − 80 °C.

For isolating total RNA, a small piece of mammary gland tissue was submerged into liquid nitrogen and grinded to a fine powder with a mortar and a pestle. Subsequently, this powder was homogenized in 1 mL TRIzol reagent (Thermo Fisher Scientific, Barcelona, Spain) with a homogenizer device (IKA T10 basic ULTRA-TURRAX, Barcelona, Spain). The Ambion RiboPure kit (Thermo Fisher Scientific, Barcelona, Spain) was used to purify total RNA in accordance with the instructions of the manufacturer. The concentration and purity of RNA preparations were evaluated with a Nanodrop ND-1000 spectrophotometer (Thermo Fisher Scientific, Barcelona, Spain), while RNA integrity was checked in a Bioanalyzer-2100 (Agilent Technologies, Santa Clara, CA) by using an Agilent RNA 6000 Nano kit (Agilent Technologies, Inc., Santa Clara, CA). The RNA integrity number (RIN) ranged between 6.00-8.40, with an average of 7.43 ± 0.58.

Paired-end sequencing (2 × 76 bp) of the RNA was performed in the Centre Nacional de Anàlisi Genòmica (CNAG-CRG, http://www.cnag.crg.eu/). The RNA-Seq library was prepared with KAPA Stranded mRNA-Seq Illumina Platforms Kit (Roche). Briefly, 500 ng total RNA were used as the input material, the poly-A fraction was enriched with oligo-dT magnetic beads and the RNA was fragmented. The strand specificity was achieved during the second strand synthesis performed in the presence of dUTP. The blunt-ended double stranded cDNA was 3’-adenylated and Illumina platform compatible adaptors with unique dual indexes and unique molecular identifiers (Integrated DNA Technologies, Coralville, IA) were ligated. The ligation product was enriched by 15 cycles of PCR amplification and the quality of the final library was validated on an Agilent 2100 Bioanalyzer with the DNA 7500 assay (Agilent Technologies, Inc., Santa Clara, CA). The libraries were sequenced with a HiSeq 4000 instrument (Illumina, San Diego, CA) in a fraction of a HiSeq 4000 PE Cluster kit sequencing flow cell lane, following the manufacturer’s protocol for dual indexing. Image analysis, base calling and quality scoring of the run were processed using the Real Time Analysis (RTA 2.7.7) tool and subsequently FASTQ sequence files were generated.

#### Bioinformatic analyses of gene expression

Sequencing quality was evaluated with the FastQC software v0.11.7 (https://www.bioinformatics.babraham.ac.uk/projects/fastqc/). Adaptors were automatically detected and removed by using the TrimGalore 0.5.0 tool (https://www.bioinformatics.babraham.ac.uk/projects/trim_galore/), and we also trimmed reads shorter than 30 bp or those with more than 5 ambiguous bases (N). We excised 15 bp from both ends of each read because sequencing errors are more frequent in these regions [13, 14]. Clean reads were aligned to the goat reference genome ARS1 [15] with HISAT2 [16] by following the pipeline reported in [17]. The counts of unambiguously mapped reads of “protein-coding” features annotated in the general feature format (GFF) file were summarized by using the featureCounts tool [18]. Differential expression analyses were subsequently carried out by using DESeq2 software [19]. Correction for multiple testing was performed with the false discovery rate (FDR) procedure reported by Benjamini and Hochberg [20]. We considered that differential expression across two time points as relevant when two conditions were met: an absolute value of log_2_ fold change (log_2_FC) > 1.5 and a *q*-value ≤0.05. Moreover, we analyzed the functional enrichment of DE genes by employing the DAVID Bioinformatics Resources 6.8 database [21, 22]. This analysis was based on human and goat background gene sets, and statistical significance was set to a *q*-value ≤0.05.

### Performance of a genome-wide association analysis for dairy traits

#### Phenotype recording

The population sampled in the current work comprised 1,023 Murciano-Granadina goats raised in 15 farms affiliated to the National Association of Murciano-Granadina Goat Breeders (CAPRIGRAN). All farms selected for this study were connected by artificial insemination. Raw records of phenotypic traits were routinely collected by CAPRIGRAN. Phenotypes under study included milk yield at 210 d (MY210), somatic cell count (SCC), fat percentage (FP), protein percentage (PP), lactose percentage (LP), dry matter percentage (DMP) and length of lactation (LOL). Phenotypes were normalized to a standard lactation of 210 d with the exception of LOL, which was not standardized. By filtering out individuals without complete phenotypic records, 822 goats remained for GWAS analyses.

#### Genotyping with the goat SNP50 BeadChip

Blood samples were collected in EDTA K3 coated vacuum tubes and stored at − 20 °C before processing. Genomic DNA was isolated by using a modified salting-out procedure [23]. Briefly, 3 mL of whole blood were centrifuged at a speed of 2,000×*g* in the presence of 4 volumes of Red Cell Lysis Solution (Tris-HCl 10 mmol/L, pH = 6.5; EDTA 2 mmol/L; Tween 20 1%). The resulting white cell pellet was lysed with 3 mL lysis buffer (Tris-HCl 200 mmol/L, pH = 8, EDTA 30 mmol/L, SDS 1%; NaCl 250 mmol/L) and proteins were degraded by using 100 μL of proteinase K (20 mg/mL). After a 3-h incubation step at 55 °C, the lysate was chilled and 1 mL of ammonium acetate 10 mol/L was added to the lysate. After 10 min of centrifugation at 2,000×*g*, the supernatant (~ 4 mL) was transferred to a new tube containing 3 mL of isopropanol 96%. Subsequently, samples were centrifuged at 2,000×*g* for 3 min. Isopropanol was removed and the DNA pellet was washed with 3 mL of ethanol 70%. After a centrifugation step at 2,000×*g* for 1 min, the DNA pellet was dried at room temperature and eluted with 1 mL of TE buffer (Tris-HCl 10 mmol/L, EDTA 1 mmol/L, pH = 8).

All goats were typed with the Goat SNP50 BeadChip (Illumina, USA) [24] according to the instructions of the manufacturer. Markers mapping to sex chromosomes, with calling rates < 90%, or with minor allele frequencies (MAF) < 0.01, or that deviated significantly from the Hardy-Weinberg expectation (*P* ≤ 1 × 10^− 6^) were filtered out. Individuals with calling rates < 90% were also excluded. By integrating available phenotypic records, 48,722 single nucleotide polymorphisms (SNPs) and 822 goats passed the filtering criteria.

#### Population structure and statistical analyses

We investigated population structure through the principal component analysis (PCA) approach implemented in the smartPCA program of the EIGENSOFT package (version 6.1.4) [25]. The proportion of the variance explained by each significant (*P* < 0.05) principal component was computed with the twstats program [26]. Association analyses were performed with the Genome-wide Efficient Mixed-Model Association (GEMMA, version 0.98) package [27] by fitting the following linear mixed model:

$$ \mathrm{Y}=\mathrm{W}\upalpha +\mathrm{x}\upbeta +\mathrm{u}+\upepsilon $$

where *Y* represents the vector of phenotypic values of the first lactation of 822 Murciano-Granadina goats; *W* is a matrix with a column of 1 s and the fixed effects, i.e. farm (15 levels), year of birth (10 levels) and litter size (5 levels); *α* is a c-vector of the corresponding coefficients including the intercept; *x* is a n-vector of marker genotypes in each individual; *β* is the effect size of the marker (allele substitution effect); *u* is a n-vector of random effects with a n-dimensional multivariate normal distribution (0, *λτ*^− 1^*K*), being *τ*^− 1^ the variance of the residual error, *λ* the ratio between the two variance components and *K* a n × n relatedness matrix derived from the 48,722 autosomal SNPs genotypes; and *ε* is a vector of errors. In this study, the GEMMA package performs likelihood ratio tests for each SNP by contrasting the alternative hypothesis (H_1_: β ≠ 0) against the null hypothesis (H_0_: β = 0). Moreover, population structure is corrected by considering the relatedness matrix, which is built by taking into account all genome-wide SNPs as a random effect. After carrying out a correction for multiple testing based on a FDR approach [20], statistical significance was set to a *q*-value ≤0.05.

We retrieved a list of protein-coding genes that mapped within the genomic boundaries (± maximum distance of linkage disequilibrium decay, i.e. 988 kb) of leading SNPs (i.e. the SNP showing the most significant association with a given trait) with the BEDTools v2.25.0 package [28]. The amount of linkage disequilibrium (LD) between adjacent SNPs was measured as the square of the correlation coefficient (r^2^) by using the “--r2” instruction implemented in PLINK v1.9 [29]. The objective of this analysis was to check whether protein-coding genes within or near quantitative traits loci (QTLs) are differentially expressed across lactation.

## Results

### An analysis of the mammary gene expression patterns across goat lactation

#### Differential expression analysis

**Fig. 1:**
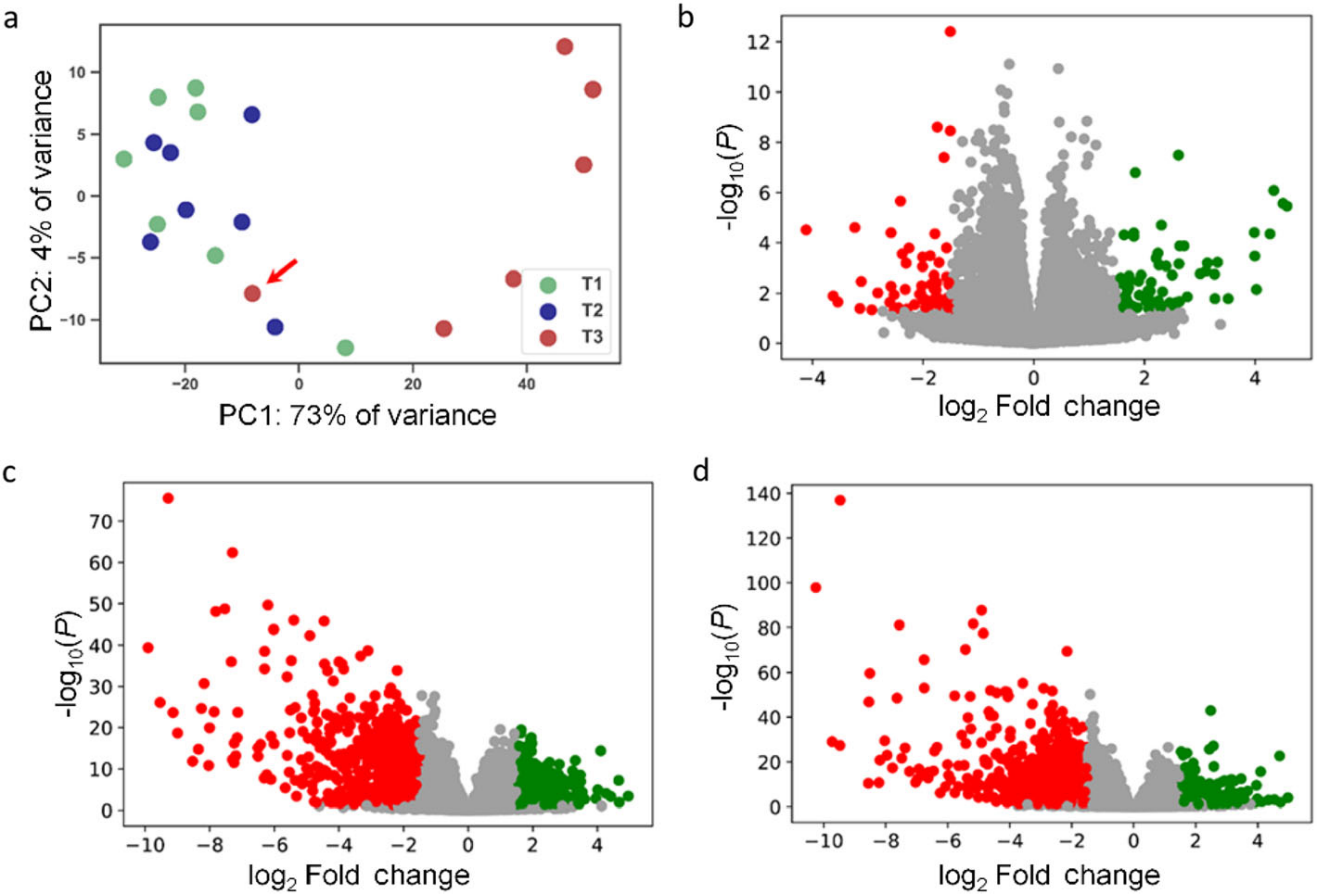
**a** Principal component analysis (PCA) of mammary samples on the basis of read counts of “protein-coding” features annotated in the general feature format (GFF) file. These samples were obtained 78 d (T1, early lactation), 216 d (late lactation, T2) and 285 d (T3, dry period) after parturition. The red arrow indicates the sample T3-22, which clusters with T1 and T2 samples probably due to an unsuccessful dry-off (Additional file 2: Figure S1). **b**-**d** Volcano plots displaying differentially expressed genes in the pairwise comparisons T1 vs. T2 (**b**), T1 vs. T3 (**c**) and T2 vs. T3 (**d**). The red and green dots denote significantly downregulated and upregulated genes, respectively

We have individually sequenced 21 RNA samples representing three lactation time points (T1, T2 and T3, see Methods). This experiment generated approximately 120 gigabases of raw data, i.e. an average of 65 million reads were obtained for each sample. The overall alignment rate obtained with HISAT2 [16, 17] was above 92%. The uniquely mapped reads were summarized by using the featureCounts tool [18]. To reduce the influence of transcriptional noise, we removed the features with a number of raw counts below 10 in all samples. Principal component analysis (Fig. 1a) based on the expression profiles of each one of the 21 samples showed a clear separation between T3 (dry period) and T1/T2 (lactation) samples. Indeed, the first component explained 73% of the total variance. The only exception was sample T3-22, which clustered with T1/T2 samples (Fig. 1a, Additional file 2: Figure S1). Our interpretation is that this sample was retrieved from a goat that was not successfully dried off, so we decided to remove it from the data set. Although T1 and T2 samples represented two different time points of lactation (Fig. 1a, Additional file 2: Figure S1), they clustered tightly.

**Fig. 2:**
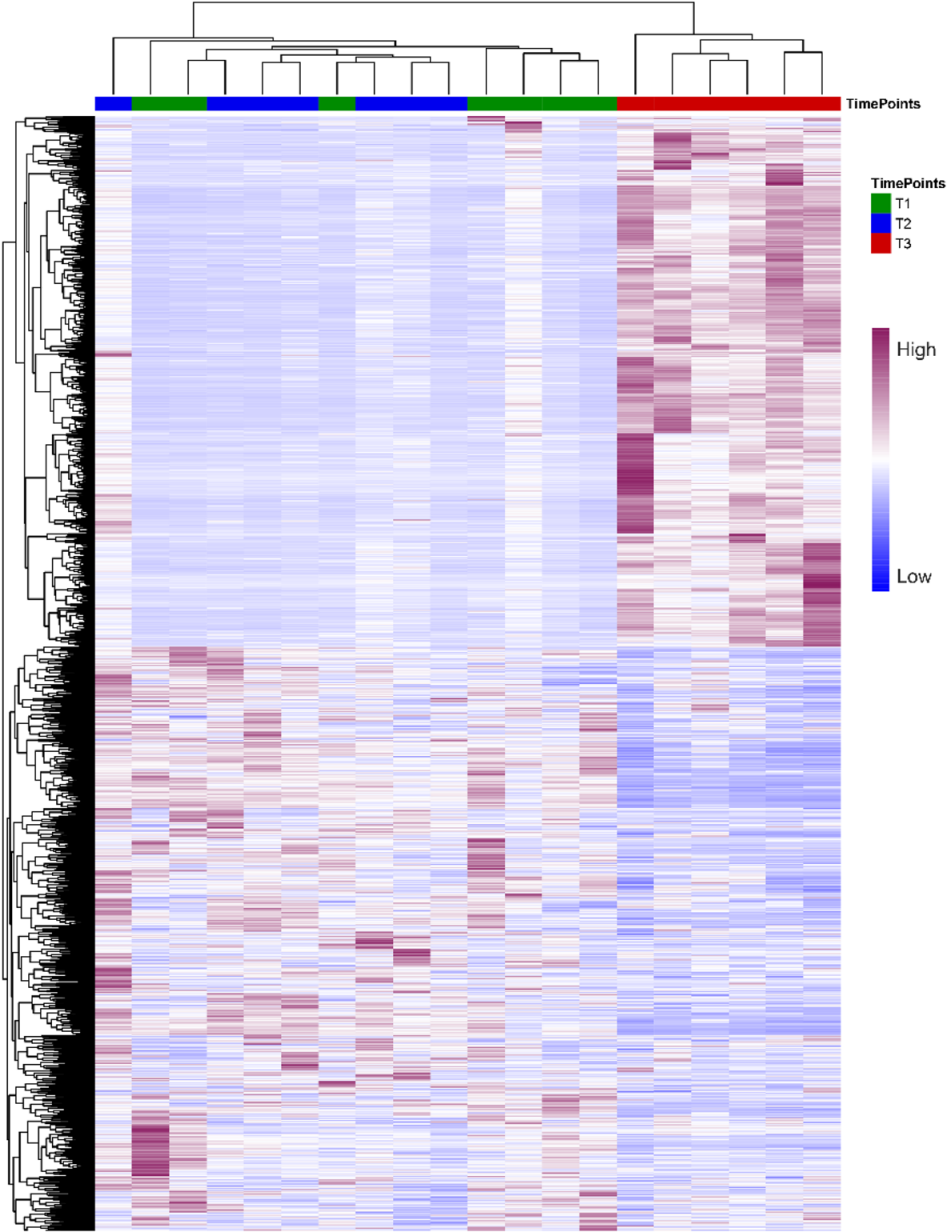
Heatmap of read counts of 1654 differentially expressed genes identified in the three available comparisons (T1 vs. T2, T1 vs. T3 or T2 vs. T3). Samples were clustered by their read counts. The color scale varying from blue to purple depicts the number of read counts of differentially expressed genes which range from low to high, respectively

A total of 16,768 genes were found to be expressed in at least one of the 20 samples corresponding to the three lactation time points (T1, T2 and T3, see Methods). By establishing as a threshold of significance a *q*-value ≤0.05 and an absolute log_2_FC > 1.5, we found 42 (T1 vs. T2), 1377 (T1 vs. T3) and 1,039 (T2 vs. T3) DE genes (Fig. 1b-d, Additional file 3: Tables S2-S4). The total set of 1,654 DE genes allowed us to differentiate T3 samples from the T1 and T2 samples (Fig. 2, Additional file 4: Figure S2). Moreover, there was a comparable number of upregulated and downregulated genes in the pairwise T1 vs. T2 (22 upregulated and 20 downregulated) and T1 vs. T3 (649 upregulated and 728 downregulated) comparisons, while in T2 vs. T3 the number of downregulated genes (695) exceeded that of upregulated genes (344) (Fig. 1b-d, Additional file 3: Tables S2-S4). In summary, our data evidenced that once lactation ceased, a large number of genes were downregulated (Fig. 1c-d, Additional file 3: Tables S3 and S4). As expected, genes encoding the main milk protein constituents such as casein α_S1_ (*CSN1S1*), casein α_S2_ (*CSN1S2*), casein β (*CSN2*), casein κ (*CSN3*), lactalbumin α (*LALBA*) and progestagen-associated endometrial protein (*PAEP*) were strongly downregulated at T3 (Table 1). The insulin receptor 1 (*IRS1*) gene, a master regulator of carbohydrate, lipid and protein metabolism, also decreased in expression at T3 (Table 1).

**Table 1:** List of differentially expressed genes mentioned in the main text

Main function	Gene symbol	T1 vs. T3		T2 vs. T3	
		log_2_FC	*q*-value	log_2_FC	*q*-value
Milk protein composition	*CSN1S1*	−8.17	1.31E-28	−8.03	8.28E-28
	*CSN1S2*	−8.00	1.83E-18	−8.18	1.50E-19
	*CSN2*	−7.24	5.06E-15	−7.23	9.68E-15
	*CSN3*	−5.16	2.76E-11	−5.12	4.67E-11
	*LALBA*	−9.53	3.72E-24	−9.47	8.10E-26
	*PAEP*	−4.67	8.16E-20	−4.41	1.61E-22
Regulator of carbohydrate, lipid and protein metabolism	*IRS1*	−1.85	1.60E-11	–	–
Fatty acid synthesis	*ACACA*	−3.10	3.26E-36	− 2.69	1.31E-39
	*FASN*	−4.46	4.34E-43	−4.06	5.25E-47
Triglyceride synthesis	*GPAM*	−5.35	5.03E-23	−5.47	2.35E-17
	*AGPAT1*	−2.18	1.66E-23	−1.96	7.68E-27
	*AGPAT4*	−1.94	2.12E-02	−2.08	8.48E-03
	*GPAT2*	–	–	−2.80	3.49E-04
	*GPAT4*	−2.70	8.24E-19	−2.44	6.98E-35
Cholesterol synthesis	*DHCR7*	−2.53	1.57E-20	−2.91	1.40E-50
	*DHCR24*	−4.00	1.15E-33	−4.14	3.81E-49
	*LSS*	−1.82	3.88E-19	−2.30	1.60E-40
	*MSMO1*	−2.47	1.01E-22	−2.93	3.50E-31
Sphingolipid synthesis	*ORMDL3*	−2.24	3.76E-19	−2.16	3.44E-30
	*OSBPL10*	−1.63	6.25E-06	–	–
	*OSBPL1A*	−1.62	4.00E-13	–	–
	*SPTLC2*	−1.73	1.56E-18	–	–
	*SPTLC3*	−1.54	2.92E-06	–	–
Acetate synthesis and fatty acid activation	*ACSS2*	−2.26	5.04E-26	−2.53	1.57E-31
	*ACSL1*	−1.98	7.21E-09	−1.97	7.05E-13
Fatty acid desaturation	*SCD*	−6.30	4.75E-32	−6.43	2.17E-23
	*FADS1*	−2.23	5.87E-26	−2.41	4.70E-25
Fatty acid absorption and transportation	*CD36*	−2.64	5.12E-16	−2.59	6.81E-20
	*LDLR*	−1.72	7.96E-08	−2.37	3.19E-20
	*FABP3*	−5.00	1.93E-09	−5.37	5.60E-12
	*APOA5*	−5.12	3.17E-07	−5.08	8.00E-08
Milk fat globules	*BTN1A1*	−6.43	1.02E-14	−6.46	5.80E-15
	*PLIN2*	−2.00	6.30E-11	−1.96	7.69E-15
	*RAB18*	−2.65	1.05E-16	−2.32	4.00E-15
	*MFGE8*	−3.84	1.67E-20	−4.09	5.24E-49
Lipolysis	*LPL*	−5.60	3.33E-30	−5.78	2.29E-47
	*LIPG*	−4.10	7.56E-11	−4.25	1.14E-11
	*PNLIPRP2*	–	–	−2.46	1.12E-02
Transcriptional regulation of lipid metabolism	*PPARA*	−1.63	1.78E-18	–	–
	*ESR2*	−1.62	8.77E-08	–	–
	*LEP*	−4.01	2.11E-04	−5.39	2.39E-08
	*INSIG1*	−4.35	1.29E-31	−5.18	9.72E-79
Transportation of carbohydrates	*SLC2A1*	−2.18	2.76E-19	−2.00	2.34E-17
	*SLC35C1*	−2.94	1.59E-22	−2.85	8.11E-23
Transportation of amino acids	*SLC1A1*	−2.14	4.08E-07	−2.01	3.14E-06
	*SLC1A5*	−2.29	3.79E-09	−2.28	5.58E-14
	*SLC7A14*	−1.83	4.38E-03	−1.84	4.00E-03
	*SLC36A2*	−1.53	2.19E-03	–	–
Transportation of minerals	*SLC30A4*	−2.70	1.37E-11	−1.95	4.49E-12
	*SLC31A1*	−1.69	1.96E-10	−1.53	6.50E-09
	*SLC39A14*	−1.72	1.31E-09	−1.68	7.69E-11
Absorption of calcium	*TRPV1*	−3.43	4.50E-06	−2.83	2.64E-07
	*TRPV5*	4.66	7.54E-07	3.68	1.15E-06
	*TRPV6*	3.25	7.87E-06	3.25	8.06E-11
	*PTHLH*	−5.17	1.04E-20	−5.41	1.28E-26
	*FGF23*	3.48	1.21E-06	2.33	5.21E-03
Apoptosis	*IGFBP5*	–	–	−1.76	2.01E-05
	*LIF*	2.58	3.05E-05	1.86	4.80E-04
	*SOCS3*	2.65	2.64E-05	2.49	1.66E-06
	*BCL2L14*	1.53	1.55E-06	1.68	4.06E-12
	*OSM*	1.90	1.46E-04	–	–
	*OSMR*	1.64	6.38E-04	1.56	6.14E-09
	*FOS*	1.67	5.82E-03	1.72	2.61E-04
	*JUNB*	2.06	4.59E-06	1.80	4.63E-11
	*TNF*	1.78	8.90E-06	–	–
	*TNFSF8*	1.66	8.87E-12	–	–
	*TNFSF13*	–	–	−2.48	3.33E-17
	*TNFRSF18*	2.10	6.21E-05	1.59	1.04E-04
	*TNFRSF6B*	1.64	6.44E-04	–	–
	*TNFAIP6*	1.81	1.41E-05	–	–
	*LTK*	−2.45	2.59E-26	−2.31	2.79E-19
	*WNT5A*	−2.50	5.73E-20	−2.28	5.87E-26
Morphogenesis and tissue remodeling	*ADAMTS4*	2.46	5.84E-05	1.61	2.64E-03
	*ADAMTS7*	1.59	2.43E-06	–	–
	*ADAMTS16*	1.69	2.46E-03	–	–
	*ADAMTS17*	−1.54	1.18E-09	−1.64	2.02E-09
Immunity	*MUC1*	−3.11	2.59E-05	−2.78	1.21E-04
	*MUC4*	−2.10	7.80E-05	−1.81	1.03E-05
	*MUC20*	−3.01	4.49E-15	−2.78	1.09E-13
	*ABCA3*	−2.61	7.95E-21	−1.94	3.98E-20
	*SFTPD*	−5.47	6.59E-34	−5.35	6.57E-38
	*BPIFA1*	−4.41	2.67E-13	−3.86	5.54E-08
	*BPIFA2*	−8.53	5.70E-11	−6.61	1.56E-14
	*BPIFA3*	−5.11	1.34E-17	−4.08	6.36E-09
	*BPIFB1*	− 4.32	3.14E-21	−4.28	3.16E-20
	*BPIFB4*	−3.93	8.81E-03	–	–
	*CLDN6*	3.09	1.68E-03	–	–
	*CLDND2*	1.68	1.57E-08	1.72	1.61E-22
Cytokines and/or their receptors	*IL5*	1.76	8.96E-03	–	–
	*IL15RA*	1.90	3.49E-07	–	–
	*IL22RA2*	1.99	9.41E-03	–	–
Defensins	*DEFB116*	2.75	2.34E-02	2.73	9.28E-03
	*DEFB126*	3.40	2.70E-03	3.55	2.21E-04
Chemokines	*CXCR4*	1.67	1.67E-05	–	–
Complement cascade	*C1QA*	1.60	1.77E-07	–	–
	*C1S*	1.69	8.27E-06	–	–
	*C1R*	1.76	4.57E-05	–	–
	*C6*	2.25	1.53E-07	1.83	2.36E-06
	*C7*	1.95	1.04E-03	–	–
	*CTSL*	1.78	8.36E-03	–	–

The dash symbol indicates the absence of a significant differential expression; log_2_FC: log_2_ of the fold-change in expression. A negative log_2_FC value indicates that mRNA expression is downregulated in T3

In T3, we also observed a marked downregulation of genes involved in lipid metabolic processes (Table 1), including: 1) Fatty acid synthesis, e.g. acetyl-CoA carboxylase α (*ACACA*) and fatty acid synthase (*FASN*); 2) Triglyceride synthesis, e.g. glycerol-3-phosphate acyltransferase, mitochondrial (*GPAM*), 1-acylglycerol-3-phosphate O-acyltransferase 1 (*AGPAT1*) and 4 (*AGPAT4*), glycerol-3-phosphate acyltransferase 2, mitochondrial (*GPAT2*) and 4 (*GPAT4*); 3) Cholesterol synthesis, e.g. 7-dehydrocholesterol reductase (*DHCR7*), 24-dehydrocholesterol reductase (*DHCR24*), lanosterol synthase (*LSS*), and methylsterol monooxygenase 1 (*MSMO1*); 4) Sphingolipid synthesis, e.g. sphingolipid biosynthesis regulator 3 (*ORMDL3*), oxysterol binding protein like 10 (*OSBPL10*) and 1A (*OSBPL1A*), serine palmitoyltransferase long chain base subunit 2 (*SPTLC2*) and 3 (*SPTLC3*); 5) Acetate synthesis and fatty acid activation, e.g. acetyl-coenzyme A synthetase 2 (*ACSS2*) and acyl-CoA synthetase long chain family member 1 (*ACSL1*); 6) Fatty acid desaturation, e.g. stearoyl-CoA desaturase (*SCD*) and fatty acid desaturase 1 (*FADS1*); 7) Fatty acid absorption and transportation, e.g. CD36 molecule (*CD36*), low-density lipoprotein receptor (*LDLR*), fatty acid binding protein 3 (*FABP3*) and apolipoprotein A5 (*APOA5*); 8) Formation of milk fat globules, e.g. butyrophilin subfamily 1 member A1 (*BTN1A1*), perilipin 2 (*PLIN2*), RAB18, member RAS oncogene family (*RAB18*), and milk fat globule-EGF factor 8 protein (*MFGE8*); 9) Lipolysis, e.g. lipoprotein lipase (*LPL*), lipase G, endothelial type (*LIPG*), and pancreatic lipase related protein 2 (*PNLIPRP2*); 10) Transcriptional regulation of lipid metabolism, e.g. peroxisome proliferator activated receptor α (*PPARA*), estrogen receptor 2 (*ESR2*), leptin (*LEP*), and insulin-induced gene 1 (*INSIG1*).

The ceasing of lactation (T3) also involved an important decrease in the gene expression of solute carrier genes (Table 1) involved in the transportation of: 1) Carbohydrates, e.g. solute carrier family 2 member 1 (*SLC2A1*) and solute carrier family 35 member C1 (*SLC35C1*); 2) Amino acids, e.g. solute carrier family 1 member 1 (*SLC1A1*), solute carrier family 1 member 5 (*SLC1A5*), solute carrier family 7 member 14 (*SLC7A14*) and solute carrier family 36 member 2 (*SLC36A2*); and 3) Minerals, e.g. zinc (solute carrier family 30 member 4, *SLC30A4*), copper (solute carrier family 31 member 1, *SLC31A1*), divalent metals (solute carrier family 39 member 14, *SLC39A14*), to mention a few. With regard to the absorption of calcium, one of the main minerals present in milk, we observed a reduction in the expression of transient receptor potential cation channel subfamily V members 1 (*TRPV1*), while there was an upregulated expression of transient receptor potential cation channel subfamily V members 5 (*TRPV5*) and 6 (*TRPV6*). The gene expression of parathyroid hormone-like hormone (*PTHLH*) was reduced in the mammary gland at T3 but, at the same time, an increased expression of fibroblast growth factor 23 (*FGF23*) was also detected.

In general, genes involved in apoptosis displayed an upregulated expression in the mammary gland of goats at T3 (Table 1). Examples of these genes are the insulin-like growth factor binding protein 5 (*IGFBP5*), leukemia inhibitory factor (*LIF*), suppressor of cytokine signaling 3 (*SOCS3*), BCL2 like 14 (*BCL2L14*), oncostatin M (*OSM*), oncostatin M receptor (*OSMR*), Fos proto-oncogene, AP-1 transcription factor subunit (*FOS*) and JunB proto-oncogene, AP-1 transcription factor subunit (*JUNB*) as well as several genes belonging to the TNF superfamily such as tumor necrosis factor (TNF) and TNF receptor superfamily members 8 (*TNFSF8*), 13 (*TNFSF13*), 18 (*TNFRSF18*) and 6b (*TNFRSF6B*), and TNF-α induced protein 6 (*TNFAIP6*). In contrast, well known survival factors such as leukocyte receptor tyrosine kinase (*LTK*) and Wnt family member 5A (*WNT5A*) displayed a reduction in their expression at T3. Moreover, several genes belonging to the family of A disintegrin and metalloproteinase with thrombospondin motifs (ADAMTS), such as *ADAMTS4*, *ADAMTS7*, *ADAMTS16* and *ADAMTS17*, which are involved in morphogenesis and tissue remodeling [30] increased in expression at T3.

With regard to genes involved in immunity, the dynamics of their expression profiles was quite heterogeneous (Table 1). Genes with key roles in mucosal immunity, e.g. mucin 1 (*MUC1*), 4 (*MUC4*) and 20 (*MUC20*), ATP binding cassette subfamily A member 3 (*ABCA3*), and surfactant protein D (*SFTPD*), were downregulated at T3. In this time point, we also detected a decreased expression of several genes, e.g. the BPI fold containing family A member 1 (*BPIFA1*), member 2 (*BPIFA2*) and member 3 (*BPIFA3*), and the BPI fold containing family B member 1 (*BPIFB1*) and member 4 (*BPIFB4*), which have antimicrobial, surfactant and immunomodulatory properties, thus preventing the formation of bacterial biofilms [31]. In contrast, tight junction proteins claudin 6 (*CLDN6*) and D2 (*CLDND2*), which determine the permeability of the paracellular barrier [32], were highly upregulated at T3.

Finally, we detected an upregulation of a broad variety of immune response genes at T3 (Table 1), including: 1) Cytokines (and/or their receptors), e.g. interleukin 5 (*IL5*), interleukin 15 receptor subunit α (*IL15RA*) and interleukin 22 receptor subunit α_2_ (*IL22RA2*); 2) Defensins, e.g. defensin β116 (*DEFB116*) and β126 (*DEFB126*); 3) Chemokines, e.g. C-X-C motif chemokine receptor 4 (*CXCR4*); and 4) Genes participating in the complement cascade, e.g. complement C1q A chain (*C1QA*), complement C1s (*C1S*), complement C1r (*C1R*), complement 6 (*C6*), complement 7 (*C7*) and cathepsin L (*CTSL*).

#### Functional enrichment of differentially expressed genes

Due to the incomplete annotation of goat genes, the functional enrichment analysis of the 1,654 DE genes was based on both human and goat background gene sets retrieved from the DAVID database [21, 22]. As a result, we identified 10 pathways that were significantly enriched based on the human background gene set (*q*-value ≤0.05, Additional file 5: Table S5), and 11 significant pathways based on the goat background gene set (*q*-value ≤0.05, Additional file 5: Table S6). Six pathways were consistently detected in both analyses, i.e. PPAR signaling, metabolic pathways, steroid biosynthesis, complement and coagulation cascades, biosynthesis of antibiotics and adipocytokine signaling (Table 2). Moreover, the gene ontology (GO) analysis based on human background genes allowed us to detect 45 significant terms, while no term was identified when the goat background genes were used (Additional file 5: Tables S5 and S6).

**Table 2:** Enriched pathways in the set of 1,654 differentially expressed genes (T1-T2, T1-T3 and T2-T3)

Name	Human background gene set				Goat background gene set			
	Number	*P* value	Fold enrichment	*q*-value	Number	*P* value	Fold enrichment	*q*-value
PPAR signaling pathway	18	2.01E-06	3.86	2.65E-05	22	8.86E-08	3.84	1.16E-06
Steroid biosynthesis	9	3.06E-05	6.46	4.03E-04	10	2.58E-05	5.63	3.38E-04
Complement and coagulation cascades	16	5.85E-05	3.33	7.70E-04	18	2.78E-05	3.19	3.65E-04
Metabolic pathways	116	1.75E-04	1.37	2.30E-03	141	6.68E-06	1.41	8.78E-05
Biosynthesis of antibiotics	28	1.49E-03	1.90	1.95E-02	30	2.58E-03	1.78	3.34E-02
Adipocytokine signaling pathway	13	2.89E-03	2.67	3.73E-02	14	3.46E-03	2.48	4.45E-02

These are the pathways that were consistently detected in the analyses based on human and goat background gene sets

### Identification of genomic regions associated with dairy traits

Descriptive statistics of seven dairy traits recorded in Murciano-Granadina goats are shown in Additional file 6: Table S7. The average values of milk fat percentage, protein percentage and milk yield normalized to 210 d were 5.20% ± 0.85%, 3.56% ± 0.41% and 387.65 ± 134.79 kg, respectively. Moreover, all traits showed a normal distribution with the exception of the somatic cell count (SCC), which was logarithmically transformed to achieve normality (Additional file 7: Figure S3). The analysis of the Murciano-Granadina individuals by PCA clustering based on the genotypes of the 48,722 available markers did not show any sign of population stratification (Additional file 8: Figure S4).

**Fig. 3:**
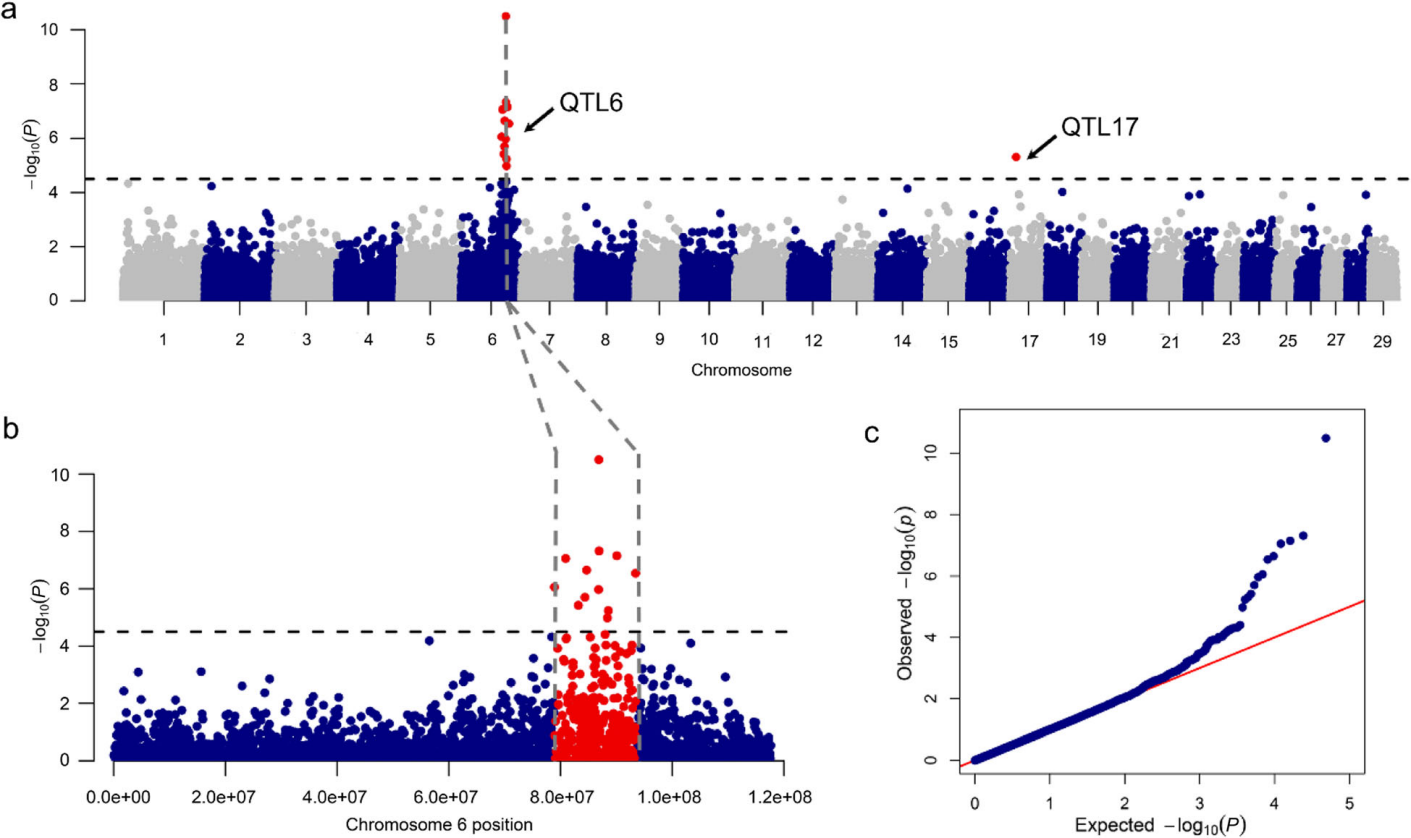
**a** Manhattan plot depicting the genome-wide association between milk protein percentage and a genomic region on chromosome 6 containing the casein genes (QTL6). Negative log_10_*P* values of the associations between SNPs and phenotypes are plotted against the genomic location of each SNP marker. Markers on different chromosomes are denoted by different colors. The dashed line represents the genome-wide threshold of significance (*q*-value ≤0.05). **b** A detailed view of the chromosome 6 region associated with protein percentage. Significant SNPs within the QTL boundaries have been marked in red. **c.** Quantile-quantile (QQ) plot of the data shown in the Manhattan plot

**Fig. 4:**
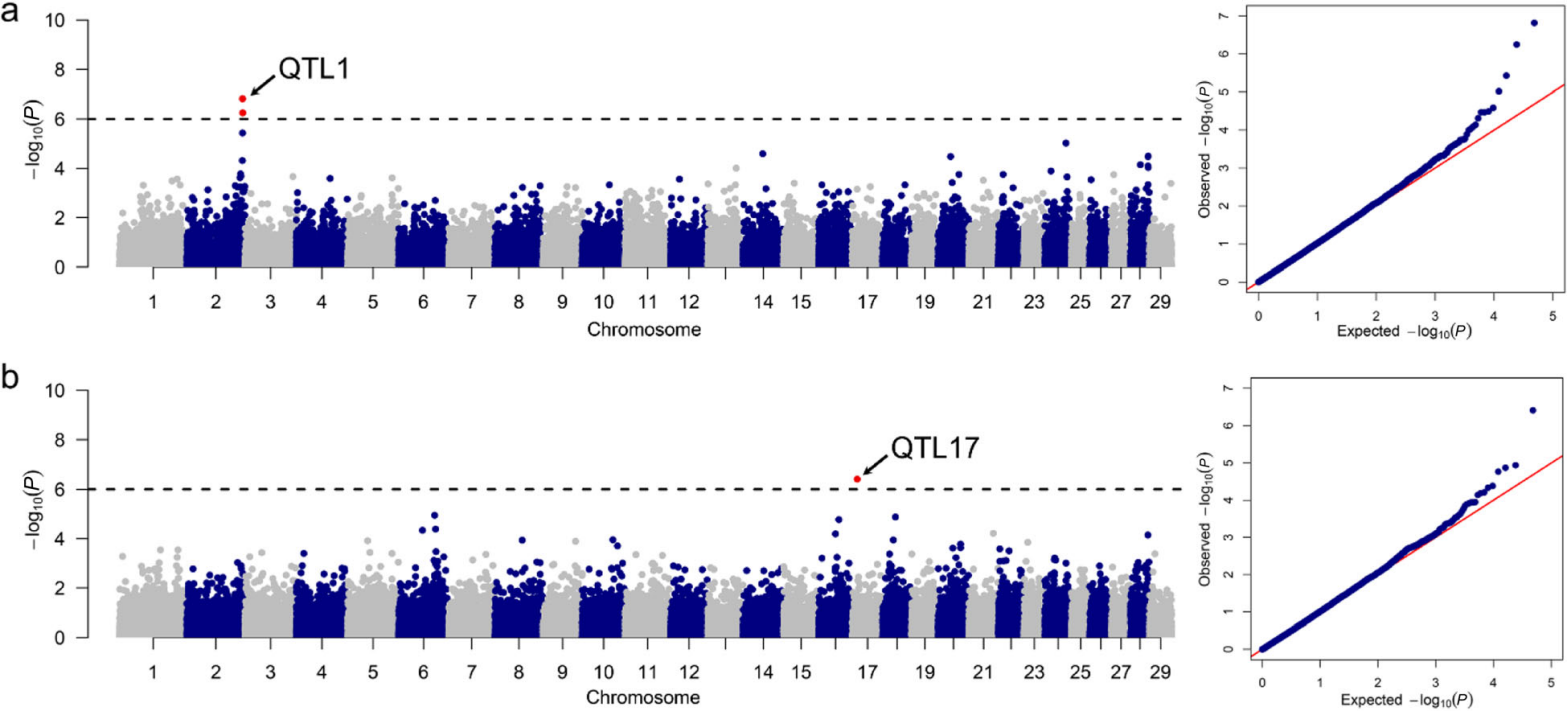
**a** Manhattan plot depicting the genome-wide significant associations between SNP markers and lactose percentage. The corresponding quantile-quantile (QQ) plot is shown at the right side of the Manhattan plot. **b** Manhattan plot depicting the genome-wide significant associations between SNP markers and dry matter percentage. The corresponding quantile-quantile (QQ) plot is shown at the right side of the Manhattan plot. Negative log_10_*P* values of the associations between SNPs and phenotypes are plotted against the genomic location of each marker SNP. Markers on different chromosomes are denoted by different colors. The dashed lines represent the genome-wide threshold of significance (*q*-value ≤0.05)

By performing association analyses between SNP genotypes and dairy traits recorded in 822 Murciano-Granadina goats, we identified 24 quantitative trait loci (QTLs) that reached the threshold of significance (*q*-value ≤0.05, Table 3) either at the genome-wide or chromosome-wide levels. Quantitative trait locus 6 (QTL6) on chromosome 6 was highly associated with protein percentage at the genome-wide level of significance (78.90-93.48 Mb, *q*-value = 1.54 × 10^− 06^*,* Fig. 3, Table 3), and also with dry matter (84.67-86.86 Mb, *q*-value = 2.66× 10^− 02^) and fat percentages (86.86 Mb, *q*-value = 1.36 × 10^− 02^) at the chromosome-wide level of significance. In addition, we found genome-wide significant associations for lactose percentage on chromosome 2 (QTL1, 130.72-131.01 Mb, *q*-value = 7.26 × 10^− 03^, Fig. 4a), as well as for protein and dry matter percentages on chromosome 17 (QTL17, 11.20 Mb, Figs. 3a and 4b). At the chromosome-wide level, we found 21 significant associations (Table 3) but only two of them were supported by more than 2 SNPs (QTL9 for somatic cell count and QTL24 for lactose percentage, Table 3).

**Table 3:** Quantitative trait loci (QTL) associated with milk traits recorded in Murciano-Granadina goats

QTL	Chromosome	Leading SNP	Position, Mb	#SNPs	MAF	Trait	β ± SE	*P* value	*q*-value
**1**	**2**	**rs268253425**	**130.72-130.01**	**2**	**0.20**	**LP**	**−0.09 ± 0.02**	**1.50E-07**	**7.26E-03**
2	3	rs268258472	113.47	1	0.38	LOL	−14.20 ± 3.21	1.04E-05	2.38E-02
3	6	rs268259784	4.40	1	0.35	PP	0.07 ± 0.02	8.21E-04	4.09E-02
4	6	rs268251267	15.64	1	0.24	PP	0.08 ± 0.03	7.85E-04	4.00E-02
5	6	rs268259390	56.51	1	0.02	DMP	1.08 ± 0.26	4.56E-05	3.56E-02
						PP	0.29 ± 0.07	6.52E-05	8.48E-03
**6**	**6**	**rs268290907**	**78.90-93.48**	**12**	**0.43**	**PP**	**−0.14 ± 0.02**	**3.19E-11**	**1.54E-06**
	6	rs268268356	84.67–86.86	2	0.40	DMP	−0.35 ± 0.08	1.14E-05	2.66E-02
	6	rs268290907	86.86	1	0.43	FP	−0.18 ± 0.04	5.79E-06	1.36E-02
7	11	rs268250457	72.83	1	0.41	LOL	15.23 ± 3.30	4.40E-06	9.13E-03
8	12	rs268256521	68.10	1	0.12	LOL	−20.35 ± 4.76	2.07E-05	3.50E-02
9	13	rs268236131	53.62-54.38	2	0.32	SCC	0.22 ± 0.05	1.92E-05	3.05E-02
11	14	rs268255959	46.10	1	0.09	LP	−0.10 ± 0.02	2.56E-05	4.77E-02
10	14	rs268282962	56.92	1	0.01	FP	0.78 ± 0.18	1.78E-05	3.32E-02
12	15	rs268235117	34.69	1	0.06	MY210	−61.98 ± 13.53	4.95E-06	7.91E-03
13	15	rs268290053	35.51	1	0.07	FP	0.35 ± 0.08	4.82E-06	7.69E-03
14	15	rs268266747	63.95	1	0.23	SCC	0.23 ± 0.05	1.63E-05	2.60E-02
15	16	rs268236985	39.59	1	0.04	DMP	0.81 ± 0.20	6.39E-05	4.93E-02
16	16	rs268253363	47.67	1	0.21	DMP	0.42 ± 0.10	1.70E-05	2.62E-02
**17**	**17**	**rs268238952**	**11.20**	**1**	**0.06**	**PP**	**0.22 ± 0.05**	**4.84E-06**	**2.13E-02**
						**DMP**	**0.88 ± 0.17**	**3.89E-07**	**1.88E-02**
18	18	rs268278435	29.64	1	0.04	DMP	0.84 ± 0.19	1.33E-05	1.70E-02
19	20	rs268277231	29.45	1	0.08	LP	−0.11 ± 0.03	3.35E-05	4.84E-02
20	22	rs268253724	25.30	1	0.36	FP	−0.18 ± 0.04	4.14E-05	4.68E-02
21	23	rs268243170	8.15	1	0.45	MY210	29.89 ± 6.76	1.03E-05	1.01E-02
22	24	rs268240589	49.71	1	0.32	LP	0.07 ± 0.02	9.51E-06	1.23E-02
23	28	rs268240830	23.02	1	0.42	LP	−0.06 ± 0.01	7.16E-05	1.66E-02
24	28	rs268246445	41.15-41.42	4	0.51	LP	−0.06 ± 0.01	3.21E-05	1.51E-02

*QTL* Quantitative trait locus; Genome-wide significant associations are indicated in bold; leading SNP: a SNP showing the most significant association with a given trait, *#SNPs* number of SNPs, *MAF* minor allele frequency, *PP* protein percentage, *FP* fat percentage, *LP* lactose percentage, *DMP* dry matter percentage, *SCC* somatic cell count, *LOL* length of lactation, *MY210* milk yield normalized to 210 d; β and SE denote the effect size of the marker (allele substitution effect) and its standard error, respectively

According to data presented in Additional file 9: Figure S5, the maximum distance at which r^2^ decays to its minimum value is 988 kb. Based on this, we retrieved 490 protein-coding genes mapping to ±988 kb of the leading SNP corresponding to each QTL. This list of genes was compared with the list of genes DE across lactation time points. By doing so, we found 39 genes mapping to 14 QTLs that are also DE (Table 4). For instance, the QTL6 region, which shows significant associations with protein, fat and dry matter percentages, contains the casein genes, which are downregulated in T3 (Tables 3 and 4).

**Table 4:** List of genes that are differentially expressed and that co-localize with dairy QTL

QTL	Leading SNP	Chromosome	Start	End	Gene symbol	T1 vs. T2		T1 vs. T3		T2 vs. T3	
						log_2_FC	*q*-value	log_2_FC	*q*-value	log_2_FC	*q*-value
1	rs268253425	2	130,227,819	130,232,923	*MSTN*	–	–	−2.17	3.30E-05	−1.66	1.14E-05
2	rs268258472	3	113,070,996	113,098,602	*SH2D1B*	–	–	1.53	3.42E-04	–	–
		3	113,497,530	113,528,836	*HSD17B7*	–	–	−2.18	1.87E-09	− 2.08	5.91E-13
		3	113,589,998	113,598,576	*CCDC190*	–	–	2.15	3.31E-02	–	–
		3	113,876,670	113,883,784	*RGS4*	–	–	−3.45	1.10E-03	−3.25	1.73E-04
4	rs268251267	6	15,922,309	15,965,996	*CFI*	–	–	2.20	4.46E-04	2.33	1.08E-05
6	rs268268356, rs268290907	6	84,458,894	84,563,377	*LOC102185449*	–	–	–	–	−2.04	2.62E-02
		6	84,667,577	84,721,849	*LOC102186288*	–	–	−4.57	7.95E-08	−3.96	5.03E-10
		6	85,137,434	85,152,951	*LOC102172432*	–	–	–	–	− 3.91	3.79E-03
		6	85,878,003	85,901,026	*LOC102169846*	–	–	−6.10	4.46E-07	−6.24	6.03E-06
		6	85,978,463	85,995,270	*CSN1S1*	–	–	−8.17	1.31E-28	−8.03	8.28E-28
		6	86,006,250	86,015,321	*CSN2*	–	–	−7.24	5.06E-15	−7.23	9.68E-15
		6	86,076,845	86,093,539	*CSN1S2*	–	–	−8.00	1.83E-18	−8.18	1.50E-19
		6	86,093,738	86,115,903	*LOC102178810*	–	–	−6.50	4.36E-12	−5.91	2.77E-11
		6	86,197,263	86,211,376	*CSN3*	–	–	−5.16	2.76E-11	−5.12	4.67E-11
		6	86,427,932	86,443,025	*AMTN*	1.94	2.72E-02	−2.59	4.31E-06	−4.56	4.61E-12
7	rs268250457	11	71,885,505	71,909,335	*GCKR*	–	–	−1.96	5.40E-09	–	–
		11	72,204,731	72,208,863	*TCF23*	–	–	2.04	3.56E-03	1.56	2.26E-02
		11	72,818,025	72,863,960	*DRC1*	–	–	2.55	2.54E-07	1.71	6.22E-04
8	rs268256521	12	67,360,683	67,392,416	*EBPL*	–	–	−2.05	6.24E-13	−2.32	7.71E-12
		12	68,130,994	68,150,636	*CYSLTR2*	–	–	1.57	1.43E-06	–	–
9	rs268236131	13	53,376,787	53,378,322	*TNFRSF6B*	–	–	1.64	6.44E-04	–	–
		13	53,475,687	53,483,660	*EEF1A2*	–	–	2.07	2.39E-02	–	–
		13	53,771,553	53,785,372	*SLC17A9*	–	–	–	–	−1.94	5.05E-10
		13	54,495,442	54,974,847	*CDH4*	–	–	1.55	1.19E-08	–	–
11	rs268255959	14	45,768,357	46,228,322	*KCNB2*	–	–	–	–	−1.63	1.45E-02
		14	46,685,533	46,688,514	*MSC*	–	–	1.58	4.90E-07	–	–
13	rs268290053	15	34,071,717	34,073,359	*LOC102175876*	4.50	3.70E-04	−4.06	9.75E-04	−8.56	7.66E-10
		15	34,118,282	34,119,849	*HBBC*	–	–	–	–	−3.72	6.30E-04
14	rs268266747	15	63,018,327	63,025,893	*C15H11orf87*	–	–	4.01	7.24E-05	–	–
15	rs268236985	16	39,165,138	39,176,197	*TNFSF18*	–	–	1.71	1.23E-02	–	–
		16	40,049,635	40,096,376	*TNFRSF8*	–	–	1.53	5.39E-04	–	–
16	rs268253363	16	47,058,035	47,175,045	*AJAP1*	–	–	2.42	2.31E-09	1.74	3.18E-05
		16	47,851,111	47,857,057	*SMIM1*	–	–	−1.53	3.69E-14	−1.52	2.29E-15
		16	47,858,258	47,874,280	*CCDC27*	–	–	−1.82	4.42E-03	–	–
		16	48,000,776	48,022,801	*LOC102183348*	–	–	1.70	1.01E-03	–	–
21	rs268243170	23	7,662,629	7,687,922	*CD83*	–	–	1.58	1.78E-06	–	–
22	rs268240589	24	49,397,175	49,422,345	*LIPG*	–	–	−4.10	7.56E-11	−4.25	1.14E-11
		24	50,316,631	50,490,273	*MAPK4*	–	–	−3.40	3.72E-15	−2.71	2.17E-12

These DE genes were retrieved from an interval of ±988 kb around leading SNPs (see Methods); Leading SNP: a SNP displaying the most significant association with a given trait; The dash symbol indicates the absence of a significant differential expression; log_2_FC: log_2_ of the fold change in expression. Start and end indicate the genomic location of the corresponding gene

## Discussion

### The expression profiles of the goat mammary gland in early and late lactation are similar

The number of DE genes in T1 vs. T2 was quite low (only 42 genes were DE), implying that the physiological and metabolic state of the mammary gland in these two time points is not remarkably different. In sheep milk, an analysis of differential expression revealed 22 (d 10 vs. 50), 20 (d 50 vs. 120), 277 (d 10 vs. 120), 135 (d 50 vs. 150) and 578 (d 10 vs. 150) DE genes [12]. The comparison that more closely resembles ours (d 50 vs. 150, 135 DE genes) highlighted a higher number of DE genes than us. Many biological and technical factors might have produced this discrepancy. For instance, we have used mammary tissue while Suárez-Vega et al. [12] employed milk somatic cells as a source of RNA. Moreover, the shape and duration of the lactation curve is different in sheep and goats. Despite these differences, a steady increase was observed in the expression of the carboxypeptidase X, M14 family member 2 (*CPXM2*) gene by Suárez-Vega et al. [12] and us. This gene might have an important role in mammary gland development and involution [12]. Moreover, Suárez-Vega et al. [12] and us observed an upregulation of the gene encoding γ-aminobutyric acid receptor subunit β_3_ (*GABRB3*) at T2, a change that has also been observed in rat lactation [33]. We have also detected an upregulation of the arylsulfatase family member I (*ARSI*), inhibin subunit β A (*INHBA*) and tenascin R (*TNR*) genes in T2, which might be indicative of the tissue remodeling and progressive involution that the mammary gland experiences through the progression of lactation [34–36]. In T2, the upregulated ST8 α-N-Acetyl-Neuraminide α-2,8-Sialyltransferase 6 (*ST8SIA6*) and polypeptide N-acetylgalactosaminyltransferase 14 (*GALNT14*) genes respectively catalyze the formation of milk sialoglycoconjugates [37] and the O-glycosylation of mucins [38]. Finally, two molecules, i.e. adiponectin (*ADIPOQ*) and hexokinase domain containing 1 (*HKDC1*), showed an increased and reduced expression in T2, respectively. These two molecules increase glucose utilization, reflecting the complex metabolic changes that the mammary gland undergoes throughout lactation.

### Remarkable differences in the mammary mRNA expression profiles of lactating and dried goats

#### The mRNA expression of genes involved in milk protein synthesis is reduced during the dry period

In contrast with the previous comparison, the gene expression profiles of the goat mammary gland are quite different when T1/T2 samples are compared to T3 samples. At T3, we have observed a 5-8 fold downregulation of the genes encoding caseins (the major protein components of milk), while there was also a 9.5-fold reduction in the gene expression of the milk whey LALBA protein, which is essential for the synthesis of lactose [39]. Likewise, the *PAEP* gene, which encodes the major whey protein β-lactoglobulin, was down-regulated 4.5-fold at T3. Similar results have also been obtained in sheep and cattle [10–12, 40]. The reduction in milk protein synthesis can be attributed to the fact that this is an energetically demanding process that is rapidly inhibited in the absence of proper hormonal and nutritional stimulation [41]. In rodents, milk protein synthesis appears to be under the control of the signal transducer and activator of transcription 5 (STAT5) factor [42], but in close similarity to what has been observed in cattle [43], we did not observe a change in the expression of the *STAT5A* or *STAT5B* genes. Conversely, there was a 2-fold reduction of the E74 like ETS transcription factor 5 (*ELF5*), which was also detected in cattle by Bionaz and Loor [43]. The *ELF5* gene is regulated by *STAT5* and induced by insulin, which might be a major player in the activation of protein synthesis in the bovine mammary gland. Furthermore, and as discussed by Bionaz and Loor [43], one of the factors that probably contributes to the strongly lowered milk protein synthesis during the dry period (T3) is the mRNA downregulation of major amino acid transporters, such as *SLC1A1*, *SLC1A5, SLC7A14* and *SLC36A2* [44–46].

#### The expression of genes involved in carbohydrate and lipid metabolism is downregulated in the dry period

Carbohydrate metabolism is also affected by the ceasing of lactation and, as mentioned before, LALBA, an enzyme necessary for the synthesis of lactose [39], the major sugar in milk, was downregulated at T3. We also observed a decrease in the mRNA expression of the *IRS1* gene, which mediates the effects of insulin [47]. Besides being fundamental for the absorption and storage of glucose [48], insulin also has important effects on the synthesis of milk proteins [49]. While the abundance of *SLC2A1* mRNA, one of the main glucose transporters, decreased at T3, we did not observe the same trend for *SLC2A4*, which is another major insulin-responsive glucose transporter [50]. These results agree with data presented by Komatsu et al. [51] who showed that SLC2A1 has a more predominant role than SLC2A4 in the glucose metabolism of the mammary gland during lactation.

The metabolic downregulation of the mammary gland that takes place during dry period has also a major impact on lipid metabolism. At T3, important transcriptional regulators were downregulated, such as *PPARA*, which is expressed in tissues with a high rate of fatty acid catabolism [52]; *ESR2*, which can inhibit ligand-mediated PPARG-transcriptional activity [53]; *LEP*, encoding a hormone that stimulates fatty acid oxidation; and *INSIG1*, encoding a protein that inhibits the proteolytic activation of sterol regulatory element-binding proteins (SREBPs). As mentioned by Bionaz and Loor [54], the case of *INSIG1* is quite counterintuitive because the mRNA expression of this gene is upregulated during lactation despite its inhibitory action on SREBPs and lipogenesis. Our interpretation is that the increased expression of *INSIG1* during lactation arises from the increased need to fine tune the activity of SREBPs. The pathway enrichment analysis also detected many biochemical routes related to lipid metabolism, including the PPAR signaling pathway. Indeed, PPARG is a master regulator of adipocyte differentiation and lipid and glucose homeostasis [55], and according to Bionaz and Loor [54], PPARG, PPARGC1A, and INSIG1, rather than SREBP1, have a pivotal role in milk fat synthesis in cattle..

#### Alterations in the expression of genes modulating calcium homeostasis

In mammals, maternal calcium homeostasis is often challenged by the high calcium demand associated with the lactation process [56]. In the epithelial mammary cell, calcium is stored in and around the Golgi apparatus, and it is secreted into milk in close association with caseins [56]. At T3, the mammary glands of Murciano-Granadina goats displayed reduced mRNA levels of *PTHLH*, a molecule that favors calcium mobilization through bone resorption during lactation [57], and in parallel, an increased mRNA expression of the *FGF23* gene, which inhibits the synthesis of parathyroid hormone [58]. We also observed an upregulation of the *TRPV5* and *TRPV6* mRNAs, which favor calcium uptake in a broad array of tissues with predominance of kidney [59] and of intestine [60], respectively. From our perspective, the increased expression of these two channels at T3 is quite paradoxical because the abrogation of lactation implies a strong reduction of the calcium demand. A possible explanation is that the increased expression of *TRPV5* and *TRPV6* genes might contribute to replenish the exhausted mammary calcium pool, but this hypothesis needs to be verified.

#### Increased mammary expression of genes related with cell death and tissue remodeling during the dry period

During the dry period (T3), there is an extensive involution, apoptosis and remodeling of the mammary gland that involves the death and replacement of senescent alveolar cells [61], transforming the udder from a milk factory to a quiescent organ [62]. Probably, one of the main cues that triggers this process is milk stasis [63]. The *FOS* and *JUNB* genes are upregulated in the mammary gland of Murciano-Granadina goats at T3, a finding that is relevant because they form part of the activator protein 1 (AP-1) dimeric transcription factor. This dimeric transcription factor is probably involved in the initiation or execution of apoptosis after mammary gland stops to milk [64]. We have also detected an increased expression of *OSM* and its receptor (*OSMR*), *LIF*, *BCL2L14*, *IGFBP5* and *SOCS3* mRNAs, a set of molecules which are known to promote the death of mammary epithelial cells and to facilitate the involution of the mammary gland [65–68]. Furthermore, metalloproteinases with aggrecanase (ADAMTS4) and cartilage oligomeric matrix protein-cleaving (ADAMTS7) activities [30] were also upregulated at T3, probably because of the extensive tissue remodeling takes place during mammary involution [69]. Indeed, metalloproteinases play a fundamental role not only in the remodeling of the epithelial ductal and vascular networks, but also in the correct synchronization of parenchymal, stromal and extracellular matrix homeostasis.

#### Complex changes in the expression of genes with immunological functions

Bacterial infections are seven times more prevalent during the early dry period than during lactation [70], thus increasing the risk to suffer mastitis in the subsequent lactation. The mammary gland can be considered as a temporal mucosal organ [71], and in this regard we have detected a downregulation, at T3, of several molecules that are involved in the synthesis of mucins (*MUC1*, *MUC4* and *MUC20*) or surfactant (*ABCA3* and *SFTPD*) substances. These are two major components of the chemical barrier that protects mucosal surfaces against bacterial infection and biofilm formation. Mucins are large O-linked glycoproteins that form part of the gel-like extracellular matrix known as mucus [72]. This is considered to be the first line of defense against pathogens because it can trap bacteria and slow down the diffusion of large viruses and, moreover, it holds immunoglobulin A and antimicrobial peptides that facilitate the elimination of pathogenic microorganisms [72]. Surfactant, which is mainly constituted by proteins and lipids, can also stimulate the clearance of microorganisms by increasing the membrane permeability of bacteria and by enhancing phagocytosis featured by cells of the innate immune system [73]. At T3, we have also detected a lowered mammary expression of the *BPIFA1*, *BPIFA2*, *BPIFA3*, *BPIFB1*and *BPIFB4* mRNAs. These molecules also play an essential role in mucosal immunity, being particularly well known the BPIFA1 protein because of its abundance in respiratory secretions, its inhibitory effect on bacterial growth and biofilm formation and its immunomodulatory properties [31]. Our results might suggest that mucous and surfactant substances that protect the mammary epithelium from infectious agents are synthesized at lower levels during the dry period, but in the absence of protein data we cannot draw firm conclusions about this matter.

In parallel, we have detected an increased mRNA expression, at T3, of several complement factors that are an important component of mucosal immunity by favoring immune bacteriolysis, neutralization of viruses, immune adherence, immunoconglutination and phagocytosis [74]. Two β-defensins (*DEFB116* and *DEFB126*) were also upregulated at T3. Defensins are cationic antimicrobial peptides that bind the negatively charged outer membranes of bacteria and kill them through a variety of mechanisms including pore formation, interference with cell wall synthesis, and prokaryotic membrane depolarization [75]. Interleukin 5, CXCR4 and specific subunits of interleukins 15 and 22 receptors also showed an increase in mRNA expression at T3. Interleukin 5 is a survival factor for B-cells and eosinophils [76], while the chemokine receptor CXCR4 is a major contributor to B-cell homeostasis and humoral immunity [77]. With regard to interleukin 15, it is a pleiotropic cytokine involved in the establishment of inflammatory and protective immune responses against invading pathogens by regulating the functions of cells belonging to both the innate and adaptive immune systems [78]. In contrast, interleukin 22 promotes the proliferation of epithelial and stromal cells, thus contributing to tissue regeneration, and also to the modulation of host defense at barrier surfaces [79].

### About the genetic determinism of dairy traits in Murciano-Granadina goats

The most significant association that we have detected in our study is that between the chromosome 6 region containing the casein genes (QTL6) and protein percentage. This result is relevant because caseins constitute ~ 80% of the total milk protein content [80]. Moreover, we have observed differential expression of the four casein genes when comparing T1/T2 vs. T3. By applying a physiological candidate gene approach, Caravaca et al. [81] found that the *CSN3* genotype is significantly associated with casein and protein contents in Murciano-Granadina goats, while the *CSN1S1* genotype did not show significant associations with protein, casein, and fat concentrations. In Norwegian goats, Hayes et al. [82] described significant associations between *CSN1S1* (protein percentage and fat kilograms) and *CSN3* genotypes (fat percentage and protein percentage) and the phenotypic variation of dairy traits. In 2016, Carillier-Jacquin and colleagues [83] reported that *CSN1S1* genotypes had a significant effect on milk yield and milk fat and protein contents in French goat breeds. Moreover, a GWAS for dairy traits in Alpine and Saanen goats detected highly significant associations between markers mapping to the casein cluster and milk protein and fat contents [1]. Indeed, we also detected a chromosome-wide significant association between QTL6 and fat percentage. The pleiotropic effects of the casein genotypes on milk protein and fat contents could be due to the fact that, in the mammary epithelial cell, the transport of proteins and lipids is coupled to a certain extent [84].

Another relevant genome-wide significant association was that between QTL1 on chromosome 2 (130.72-131.01 Mb) and lactose percentage. This region overlaps the NGFI-A binding protein 1 (*NAB1*) gene, also known as EGR1 binding protein 1 gene. This gene shows an increased expression during mouse lactation and encodes a molecule that binds to the proximal promoter of the galactokinase gene, which is involved in galactose catabolism [85]. We also identified a third genome-wide significant association between a chromosome 17 region (QTL17, 11.20 Mb) and protein and dry matter percentages. This region closely maps to the T-Box 3 (*TBX3*) gene, which is highly expressed in luminal cells during early mammary gland initiation by interacting with Wnt and fibroblast growth factor (Fgf) signaling [86, 87].

The comparison of the genome-wide and chromosome-wide significant associations detected by us vs. those reported by Martin et al. [1] revealed a low level of positional concordance, suggesting the existence of a remarkable level of genetic heterogeneity amongst caprine breeds with regard to the genetic determinism of milk traits. Indeed, in the GWAS carried out by Martin et al. [1] more than 50% of the associations were exclusively detected in one of the two breeds under analysis (Alpine and Saanen) despite their close genetic relatedness. This finding supports the proposal of using breed-specific reference genomes to increase the accuracy of genomic analyses [88]. Moreover, in humans a large amount of variants occurs at different frequencies in different populations, having variable effects on complex traits and producing a substantial level of genetic heterogeneity [89]. Technical and experimental factors related to population size and marker density may also influence statistical power to detect associations [90]. Many of the QTLs detected by us were represented by a single SNP, possibly due to the low LD between nearby markers [91–94]. Finally, only a few genes located within or close to QTLs showed differential expression between T1/T2 and T3, suggesting that the set of DE genes in these two physiological states has a weak correspondence with the set of genes influencing the quantitative variation of milk traits.

## Conclusions

The ceasing of lactation in Murciano-Granadina goats involves the downregulation of the mRNA expression of many genes related to the synthesis, uptake and transportation of proteins, lipids and carbohydrates as well as changes in the mRNA expression of genes involved in the maintenance of calcium homeostasis. We also observed an increased expression of genes modulating cell death and tissue remodeling that probably mediate the involution and regeneration of the mammary gland during the dry period. From an immunological perspective, genes that contribute to the formation of mucous and surfactant barriers are downregulated in the dry period, possibly increasing the risk of infection. However, we have also observed an increase in the mRNA expression of defensin, cytokine and complement genes which should ensure the elicitation of an effective immune response against pathogens. Finally, the results obtained in the GWAS allows us to conclude that the casein genes, which are strongly downregulated during the dry period, are major genetic determinants of the phenotypic variance of milk protein and fat composition traits recorded in Murciano-Granadina goats, thus supporting the use of casein genotypes as a source of information to improve these two phenotypes.

## Supplementary information

**Additional file 1: Table S1.** Information about the Murciano-Granadina goats sampled in the RNA-Seq experiment.

**Additional file 2: Figure S1.** Similarity matrix of samples used for detecting differentially expressed genes. T1, T2 and T3 correspond to 78.25 ± 9.29 d (T1, early lactation), 216.25 ± 9.29 d (T2, late lactation) and 285.25 ± 9.29 d (T3, dry period) after parturition, respectively. The sample T3-22 (red arrow) clustered with T1/T2 samples probably because it was obtained from a goat that was not successfully dried-off at the time of sampling.

**Additional file 3: Tables S2-S4.** List of differentially expressed genes between the T1 and T2, T1 and T3, and T2 and T3 time points.

**Additional file 4: Figure S2.** Venn diagram depicting the overlaps of differentially expressed genes between pair-wise T1 vs. T2, T1 vs. T3 and T2 vs. T3 comparisons. T1 and T2 represent early (78.25 ± 9.29 d after parturition) and late (216.25 ± 9.29 d) lactation, respectively, while T3 (285.25 ± 9.29 d) corresponds to the dry period.

**Additional file 5: Table S5-S6.** Pathways and gene ontology (GO) terms enriched in the set of 1,654 differentially expressed genes on the basis of human and goat background gene sets.

**Additional file 6: Table S7.** Descriptive statistics of seven dairy traits recorded in the first lactation of 822 Murciano-Granadina goats.

**Additional file 7: Figure S3.** Histograms of the phenotypic values of the percentage of protein (a), fat (b), lactose (c) and dry matter (d), milk yield normalized to 210 d (e), length of lactation (f), logarithmically transformed somatic cell count (g) recorded in the first lactation of Murciano-Granadina goats. The raw somatic cell count is (× 10^3^ cells/mL) shown in (h).

**Additional file 8: Figure S4.** Structure of the Murciano-Granadina population employed in the GWAS as assessed by principal component analysis (PCA) based on Goat SNP50 BeadChip genotypes. PC1 and PC2 indicate the principal components 1 and 2, respectively. Values in parentheses reflect the percentage of variance in the data explained by each principal component.

**Additional file 9: Figure S5.** Linkage disequilibrium (LD) decay in 822 Murciano-Granadina goats with available Goat SNP50 BeadChip genotypes. The scatter plot shows the decline of r^2^ between single nucleotide polymorphisms (y-axis) with distance expressed in bp (x-axis). The fitting line is depicted in red.

## Data Availability

Goat SNP50 BeadChip genotypes and mammary RNA-Seq data are accessible at Figshare (https://doi.org/10.6084/m9.figshare.11881617) and Sequence Read Archive (SRA) database (PRJNA607923), respectively.

## Abbreviations

DE: Differentially expressed
DMP: Dry matter percentage
FDR: False discovery rate
FP: Fat percentage
GFF: General feature format
GO: Gene ontology
GWAS: Genome-wide association study
LD: Linkage disequilibrium
log_2_FC: log_2_ of the fold-change
LOL: Length of lactation
LP: Lactose percentage
MAF: Minor allele frequency
Mb: Megabase
MY210: Milk yield at 210 days
PCA: Principal component analysis
PP: Protein percentage
QTL: Quantitative trait locus
RNA-Seq: RNA sequencing
SCC: Somatic cell count
SNP: Single nucleotide polymorphism
