# Supplementary material for: Analyzing the genomic and transcriptomic architecture of milk traits in Murciano-Granadina goats
Source: J Anim Sci Biotechnol. 2020 Mar 11;11:35. doi: 10.1186/s40104-020-00435-4 (PMC7065321; doi:10.1186/s40104-020-00435-4)
Supplement: Supplementary file 1 — Additional file 1: Table S1. Information about the Murciano-Granadina goats sampled in the RNA-Seq experiment. [file 40104_2020_435_MOESM1_ESM.docx]

**Additional file 1: Table S1** Information about the Murciano-Granadina goats sampled in the RNA-Seq experiment

| **Goat ID** | **Birth year** | **Age**  **(years)** | **Parturition date** | **No. of lactation** | **T1**  **(20-Dec-17)** | **T2**  **(7-May-18)** | **T3**  **(15-Jul-18)** |
| --- | --- | --- | --- | --- | --- | --- | --- |
| 22 | 2009 | 8 | 2-Oct-17 | 7 | + | + | + |
| 27 | 2009 | 8 | 26-Sep-17 | 7 | + | + | + |
| 32 | 2009 | 8 | 2-Oct-17 | 7 | + | + | + |
| 44 | 2011 | 6 | 23-Sep-17 | 5 | + | + | + |
| 54 | 2012 | 5 | 17-Oct-17 | 4 | + | + | + |
| 67 | 2013 | 4 | 27-Sep-17 | 2 | - | - | + |
| 73 | 2013 | 4 | 28-Sep-17 | 2 | + | + | - |
| 74 | 2013 | 4 | 17-Oct-17 | 3 | + | + | + |

+: sampled; -: not sampled.
