# Supplementary material for: Analyzing the genomic and transcriptomic architecture of milk traits in Murciano-Granadina goats
Source: J Anim Sci Biotechnol. 2020 Mar 11;11:35. doi: 10.1186/s40104-020-00435-4 (PMC7065321; doi:10.1186/s40104-020-00435-4)
Supplement: Supplementary file 2 — Additional file 2: Figure S1. Similarity matrix of samples used for detecting differentially expressed genes. T1, T2 and T3 correspond to 78.25 ± 9.29 d (T1, early lactation), 216.25 ± 9.29 d (T2, late lactation) and 285.25 ± 9.29 d (T3, dry period) after parturition, respectively. The sample T3-22 (red arrow) clustered with T1/T2 samples probably because it was obtained from a goat that was not successfully dried-off at the time of sampling. [file 40104_2020_435_MOESM2_ESM.pptx]

## Slide 1
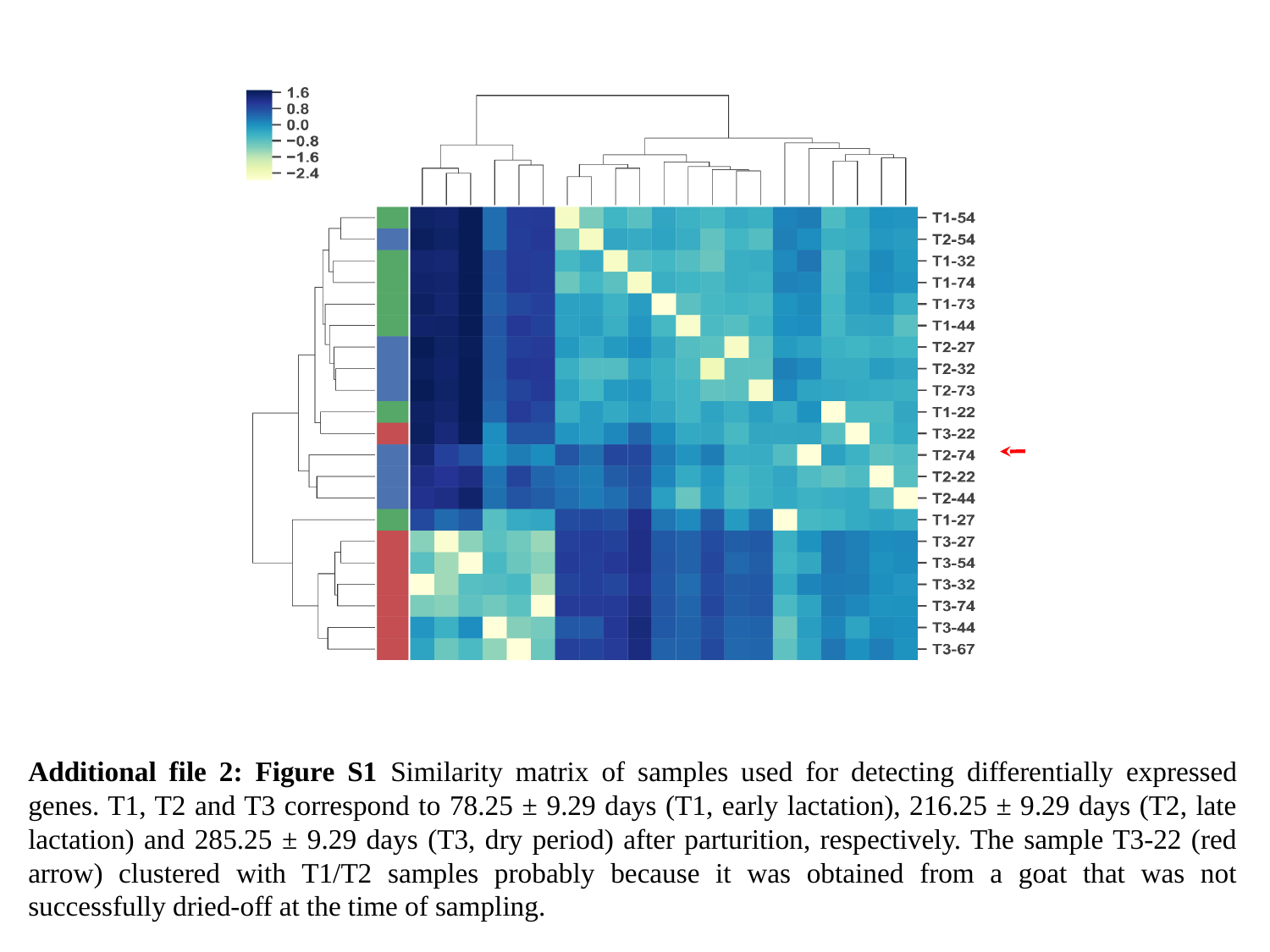

Additional file 2: Figure S1 Similarity matrix of samples used for detecting differentially expressed genes. T1, T2 and T3 correspond to 78.25 ± 9.29 days (T1, early lactation), 216.25 ± 9.29 days (T2, late lactation) and 285.25 ± 9.29 days (T3, dry period) after parturition, respectively. The sample T3-22 (red arrow) clustered with T1/T2 samples probably because it was obtained from a goat that was not successfully dried-off at the time of sampling.
