# Supplementary material for: Analyzing the genomic and transcriptomic architecture of milk traits in Murciano-Granadina goats
Source: J Anim Sci Biotechnol. 2020 Mar 11;11:35. doi: 10.1186/s40104-020-00435-4 (PMC7065321; doi:10.1186/s40104-020-00435-4)
Supplement: Supplementary file 4 — Additional file 4: Figure S2. Venn diagram depicting the overlaps of differentially expressed genes between pair-wise T1 vs. T2, T1 vs. T3 and T2 vs. T3 comparisons. T1 and T2 represent early (78.25 ± 9.29 d after parturition) and late (216.25 ± 9.29 d) lactation, respectively, while T3 (285.25 ± 9.29 d) corresponds to the dry period. [file 40104_2020_435_MOESM4_ESM.pptx]

## Slide 1
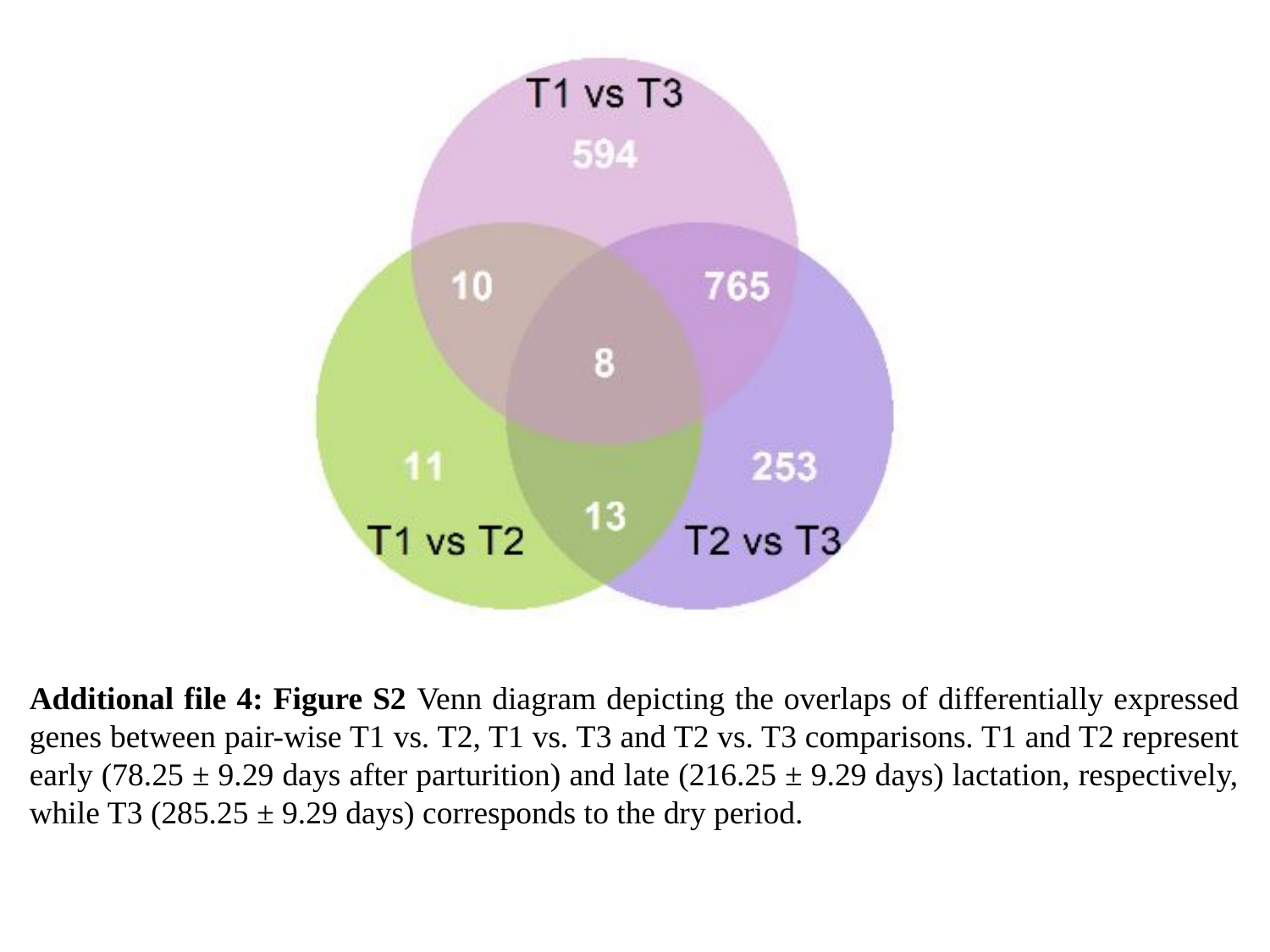

Additional file 4: Figure S2 Venn diagram depicting the overlaps of differentially expressed genes between pair-wise T1 vs. T2, T1 vs. T3 and T2 vs. T3 comparisons. T1 and T2 represent early (78.25 ± 9.29 days after parturition) and late (216.25 ± 9.29 days) lactation, respectively, while T3 (285.25 ± 9.29 days) corresponds to the dry period.
