# Supplementary material for: Analyzing the genomic and transcriptomic architecture of milk traits in Murciano-Granadina goats
Source: J Anim Sci Biotechnol. 2020 Mar 11;11:35. doi: 10.1186/s40104-020-00435-4 (PMC7065321; doi:10.1186/s40104-020-00435-4)
Supplement: Supplementary file 7 — Additional file 7: Figure S3. Histograms of the phenotypic values of the percentage of protein (a), fat (b), lactose (c) and dry matter (d), milk yield normalized to 210 d (e), length of lactation (f), logarithmically transformed somatic cell count (g) recorded in the first lactation of Murciano-Granadina goats. The raw somatic cell count is (× 103 cells/mL) shown in (h). [file 40104_2020_435_MOESM7_ESM.pptx]

## Slide 1
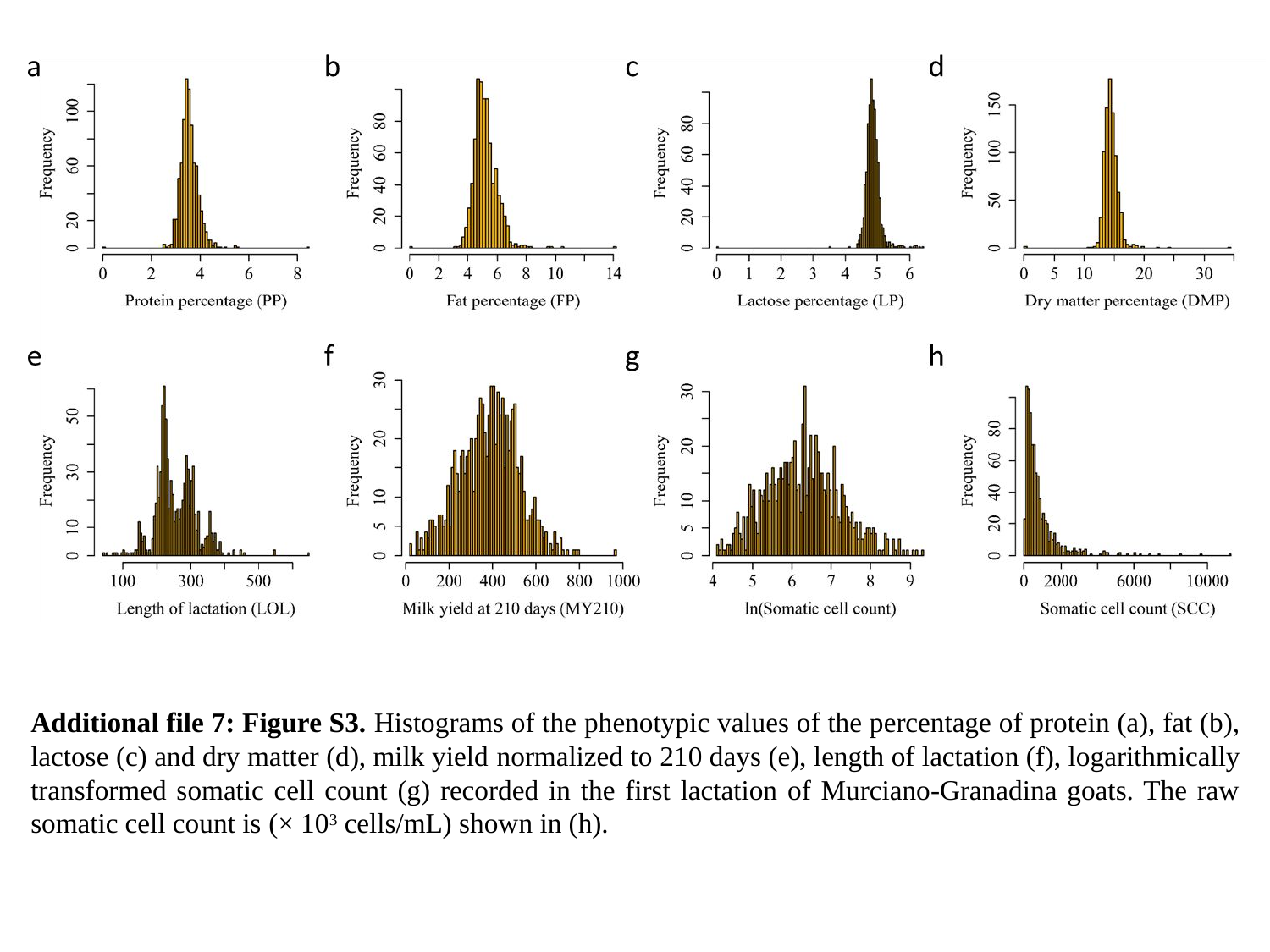

Additional file 7: Figure S3. Histograms of the phenotypic values of the percentage of protein (a), fat (b), lactose (c) and dry matter (d), milk yield normalized to 210 days (e), length of lactation (f), logarithmically transformed somatic cell count (g) recorded in the first lactation of Murciano-Granadina goats. The raw somatic cell count is (× 103 cells/mL) shown in (h).
