# Supplementary material for: Analyzing the genomic and transcriptomic architecture of milk traits in Murciano-Granadina goats
Source: J Anim Sci Biotechnol. 2020 Mar 11;11:35. doi: 10.1186/s40104-020-00435-4 (PMC7065321; doi:10.1186/s40104-020-00435-4)
Supplement: Supplementary file 8 — Additional file 8: Figure S4. Structure of the Murciano-Granadina population employed in the GWAS as assessed by principal component analysis (PCA) based on Goat SNP50 BeadChip genotypes. PC1 and PC2 indicate the principal components 1 and 2, respectively. Values in parentheses reflect the percentage of variance in the data explained by each principal component. [file 40104_2020_435_MOESM8_ESM.pptx]

## Slide 1
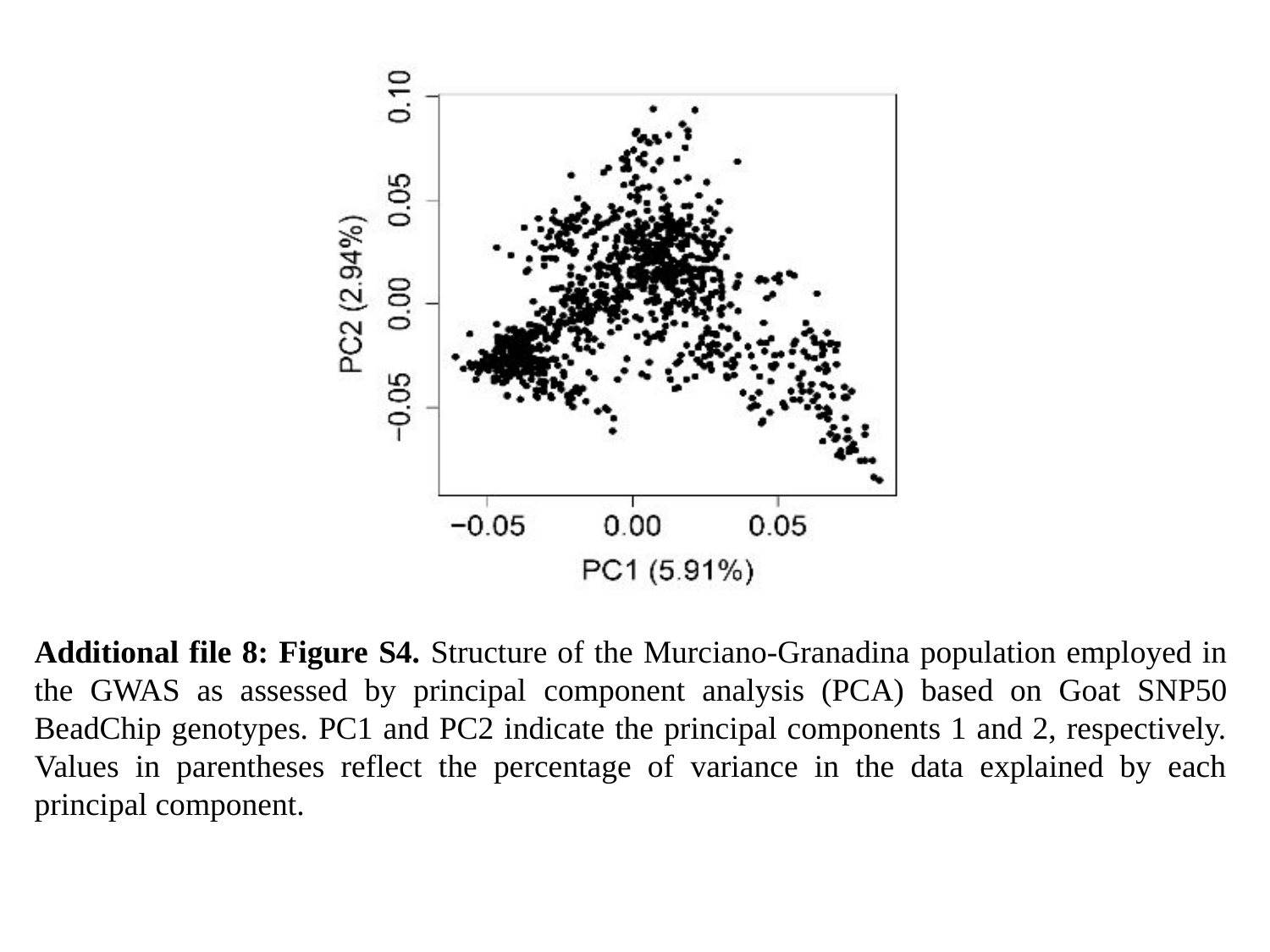

Additional file 8: Figure S4. Structure of the Murciano-Granadina population employed in the GWAS as assessed by principal component analysis (PCA) based on Goat SNP50 BeadChip genotypes. PC1 and PC2 indicate the principal components 1 and 2, respectively. Values in parentheses reflect the percentage of variance in the data explained by each principal component.
