# Supplementary material for: Analyzing the genomic and transcriptomic architecture of milk traits in Murciano-Granadina goats
Source: J Anim Sci Biotechnol. 2020 Mar 11;11:35. doi: 10.1186/s40104-020-00435-4 (PMC7065321; doi:10.1186/s40104-020-00435-4)
Supplement: Supplementary file 9 — Additional file 9: Figure S5. Linkage disequilibrium (LD) decay in 822 Murciano-Granadina goats with available Goat SNP50 BeadChip genotypes. The scatter plot shows the decline of r2 between single nucleotide polymorphisms (y-axis) with distance expressed in bp (x-axis). The fitting line is depicted in red. [file 40104_2020_435_MOESM9_ESM.pptx]

## Slide 1
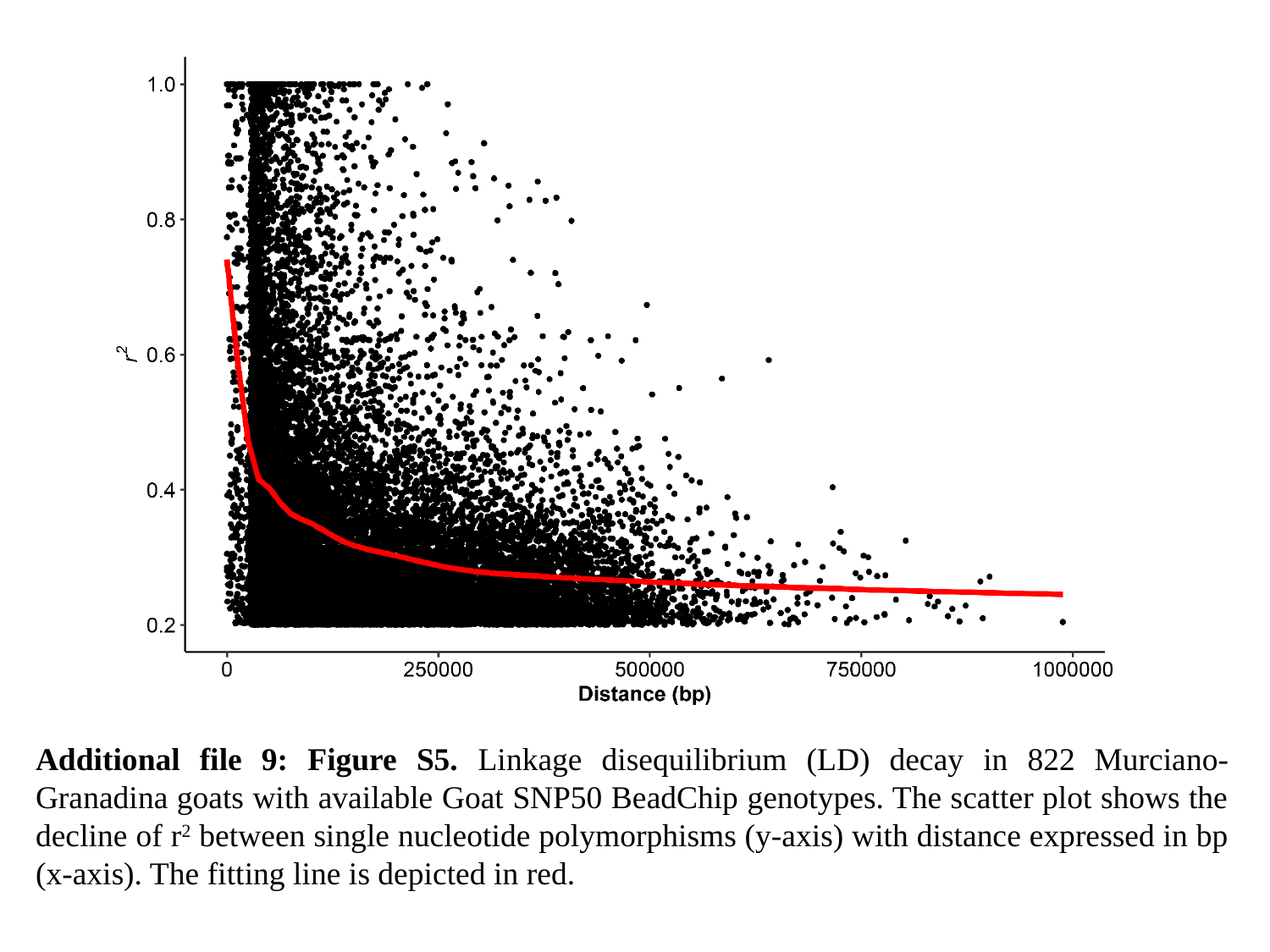

Additional file 9: Figure S5. Linkage disequilibrium (LD) decay in 822 Murciano-Granadina goats with available Goat SNP50 BeadChip genotypes. The scatter plot shows the decline of r2 between single nucleotide polymorphisms (y-axis) with distance expressed in bp (x-axis). The fitting line is depicted in red.
